# Unravelling seasonal trends in coastal marine heatwave metrics across global biogeographical realms

**DOI:** 10.1038/s41598-022-11908-z

**Published:** 2022-05-11

**Authors:** François Thoral, Shinae Montie, Mads S. Thomsen, Leigh W. Tait, Matthew H. Pinkerton, David R. Schiel

**Affiliations:** 1 Marine Ecology Research Group and Centre for Integrative Ecology, School of Biological Sciences, University of Canterbury, Christchurch, New Zealand; 2 NIWA, Christchurch, New Zealand; 3 NIWA, Wellington, New Zealand

**Keywords:** Marine biology, Biogeography, Biooceanography, Biogeography, Biooceanography

## Abstract

Marine heatwaves (MHWs) can cause dramatic changes to ecologically, culturally, and economically important coastal ecosystems. To date, MHW studies have focused on geographically isolated regions or broad-scale global oceanic analyses, without considering coastal biogeographical regions and seasons. However, to understand impacts from MHWs on diverse coastal communities, a combined biogeographical-seasonal approach is necessary, because (1) bioregions reflect community-wide temperature tolerances and (2) summer or winter heatwaves likely affect communities differently. We therefore carried out season-specific Theil–Sen robust linear regressions and Pettitt change point analyses from 1982 to 2021 on the number of events, number of MHW days, mean intensity, maximum intensity, and cumulative intensity of MHWs, for each of the world’s 12 major coastal biogeographical realms. We found that 70% of 240 trend analyses increased significantly, 5% decreased and 25% were unaffected. There were clear differences between trends in metrics within biogeographical regions, and among seasons. For the significant increases, most change points occurred between 1998 and 2006. Regression slopes were generally positive across MHW metrics, seasons, and biogeographical realms as well as being highest after change point detection. Trends were highest for the Arctic, Northern Pacific, and Northern Atlantic realms in summer, and lowest for the Southern Ocean and several equatorial realms in other seasons. Our analysis highlights that future case studies should incorporate break point changes and seasonality in MHW analysis, to increase our understanding of how future, more frequent, and stronger MHWs will affect coastal ecosystems.

## Introduction

Marine heatwaves (MHWs) are discrete and prolonged anomalously warm water events that have caused major changes to marine ecosystems and the services they provide^1,2^. MHWs across the globe are becoming longer, more frequent, and more intense^1,3–5^. This trend is expected to accelerate under future climate change, potentially pushing many marine organisms and ecosystems to the limits of their resilience^3^. Studies on MHWs have increased dramatically, partly because impacts are more severe, but also because metrics and computer code have been developed to describe MHWs (such as frequency, mean, maximum and cumulative intensity^6^) and long-term satellite-based sea surface temperature data are freely available^7^. Where biological baseline data exist, the metrics, code and satellite data allow researchers to do retrospective impact-analyses of past MHWs to better understand their legacy effects. To date, most MHW studies have focused on unique events and specific geographical case studies^8–13^ or on global analysis of oceanic regions that are dominated by sparse pelagic mobile organisms and deep-water pelagic ecosystems with relatively low diversity and productivity^3,4,14–16^. Indeed, changes to MHWs in surface waters may have few cascading impacts on deep water animal communities. By contrast, global MHW analyses have rarely focused on shallow coastal waters where biodiversity and productivity are much higher and where changes to these processes are likely to be more dramatic, nor have they compared trends between biogeographical regions or seasons. Indeed, the greatest ecological effects from MHWs have been documented on highly productive coastal marine foundation species, including kelps, seagrasses and corals, and their associated diverse communities^17–20^. In addition to well-studied kelp, seagrass and coral habitats, coastal bioregions also support highly diverse and productive oyster and mussel beds, mangroves, salt marshes, tubeworm gardens^21^ and sponge beds^22,23^. Furthermore, coastal ecosystems face significant co-occurring stressors, like eutrophication, invasions, high fishing pressure and increased turbidity that may exacerbate MHW impacts^24^. To understand how MHWs affect coastal areas with high biodiversity and critical foundation species, targeted analyses are required, as argued by Marin et al.^25^ who assessed changes over 25 years to MHWs in coastal areas but without considering seasonal effects across bioregions.

It is important to analyse trends in MHWs in a biogeographical context because bioregions delineate ecosystems and communities with shared adaptations to a local environment. Ambient temperature conditions and temperature tolerances of key species are particularly important in defining these boundaries^22^. Finally, MHWs should be analysed across different seasons because survival, growth, reproduction, and species interactions vary over a year in response to cyclic changes in solar radiation, daylength and temperature^26^; MHWs can disrupt seasonal growth and may exceed species-specific thresholds during different seasons^27^. Most research has analysed summer MHWs^8,28,29^ or has pooled temperature data across seasons^5,14,25^. However, MHWs can also occur in winter, spring or autumn^30^, potentially causing different biological effects due to seasonal acclimation and changing baseline conditions^27^. For example, La Sorte et al.^16^ recently analysed seasonal variation in air temperature across terrestrial and marine systems, documenting how the frequency and duration of extreme heating events changed across summer and winter. There are, however, important differences in effects from air and sea temperatures, in part because of their different capacities to store and transfer heat and resulting, for example, in more variable daily air temperature^31^. Although intertidal communities are subjected to both sea and air temperatures at times, the analysis of La Sorte et al.^16^ is less suitable to understand changing temperature stress on subtidal communities.

Here we built on the aforementioned two recent studies that analysed changes to MHWs along coastlines^25^ and seasons^16^. Our objectives were to use robust linear regressions^32,33^ to quantify the severity (from significance tests and regression slopes) and timing (temporal change points) of MHWs across metrics, seasons, and coastal biogeographic realms.

## Results

### General trends across seasons and biogeographical realms

**Figure 1:**
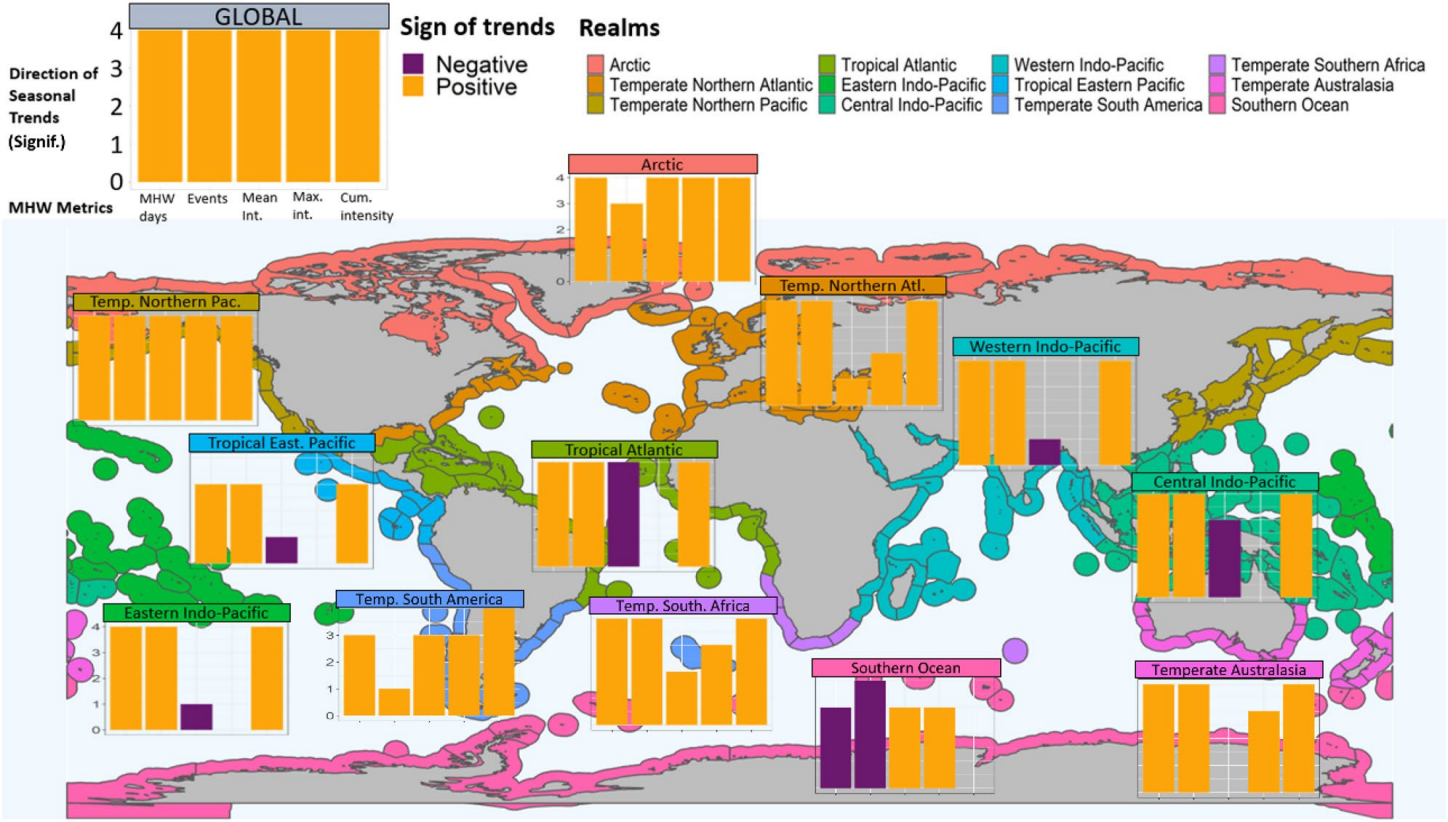
Map showing the direction of trend from seasonal analyses on MHW days, number of events, mean intensity, maximum intensity, and cumulative intensity (bars from left to right; 4 seasonal analyses per metric) at a global scale (insert, 260,010 pixels) and for 12 coastal realms (map)^22^. Of the 240 analyses (12 regions × 5 metrics × 4 seasons, see Figs. 3, 4, 5, 6), 70% increased significantly, 5% decreased and 25% were unaffected. Map and figures were generated using the R free software environment (version 4.1.0, https://www.r-project.org/).

**Figure 2:**
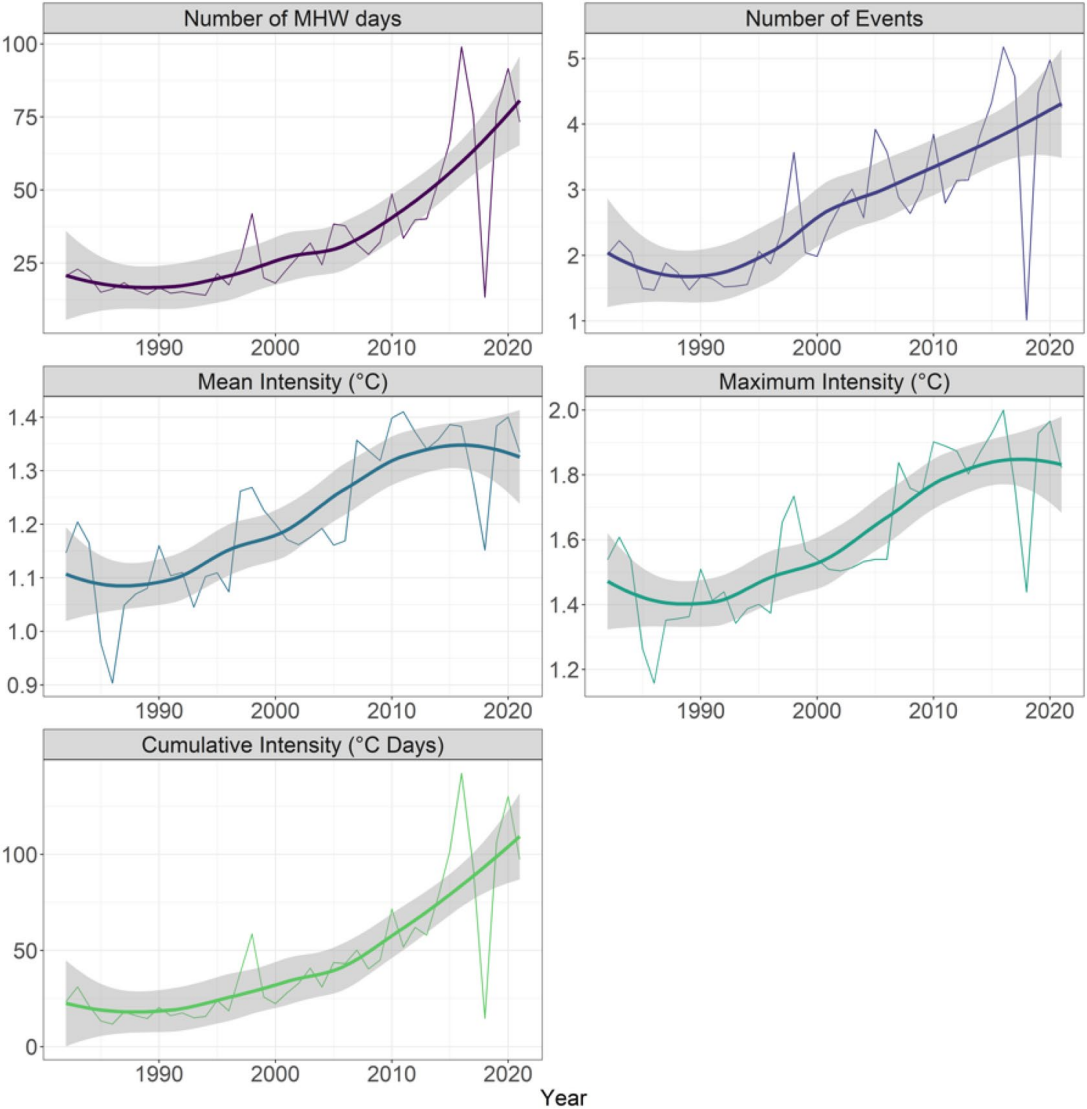
Global trends from January 1982 to December 2021 in the number of yearly-averaged and pixel-averaged marine heatwave days, number of events, mean intensity (°C), maximum intensity (°C) and cumulative intensity (°C days) of marine heatwaves (thin lines). Thick lines show loess smoothing ± 95% confidence intervals. See Table S1 for specific trendlines, *p* values and break point years.

Globally and along the coastal zone, MHWs show positive and significant trends in five key metrics across all seasons between 1982 and 2021 (Fig. 1). MHW days almost tripled from ca. 25 to 75 days on average per year per pixel, and the frequency of MHWs doubled from ca. 2 to 4 events on average per year per pixel. Similarly, the averaged mean and maximum intensity increased from ca. 1.1 to 1.3 and 1.5 to 1.8 °C per year per pixel, respectively. As a result, the cumulative intensity has more than quadrupled from ca. 25 to 100 °C days (Fig. 2). Mann–Kendall and Theil–Sen’s analyses showed that increasing trends were significant over the 4 decades. Significant change points were detected for MHW days, the number of events and cumulative intensity since 2001. Similarly, mean and maximum intensities intensified in 1996 and 2004, respectively (although not significantly so, Table S1). When the trend analyses were separated into before and after change point detection, much more severe intensification was found for all five metrics, being 20.8 times higher for MHW days, 4.7 times higher for MHW events, 9.9 times higher for mean intensity, 1.9 times higher for maximum intensity, and 7.3 times higher for cumulative intensity after the change point year (typically early 2000s).

Similar patterns were found across seasons where analyses again showed significant positive trends for all MHW metrics for individual seasons (Figure S1, Table S2). Significant change point detections showed that MHWs over summer months were more frequent for number of days (since 2002) and events (since 2001), and more severe for mean and maximum intensity (since 2003), and cumulative intensity (since 2002). In autumn, MHWs were more frequent since 2001 (days) and 2000 (events) and more intense since 2005 (mean), 2004 (maximum) and 2002 (cumulative). In winter, MHWs were more frequent since 2004 (days) and 2001 (events) and more intense since 1996 (for all three intensity metrics). Finally, in spring, MHWs were more frequent since 2001 (days) and 1996 (events) and more intense since 2006 (mean and maximum) and 1996 (cumulative) (Table S2). Significant decadal trends in metrics for the period after change point detection showed that the largest increase in trends for MHW days occurred in winter (9.5 more days per decade per pixel = 18.1 times higher than before 2004) and for number of events, where the largest trend increase occurred in autumn (0.34 more events per decade per pixel = 6.4 times higher than before 2000). By contrast, trends in maximum (0.25 °C more per decade per pixel = 5.7 times higher than before 2003) and cumulative intensity (13.791 °C days more per decade per pixel = 8.1 times higher than before 2002) were highest in summer after the change point year (Table S2).

MHWs also affected the coastal biogeographical realms differently. For example, the Arctic realm was most and the Southern Ocean least affected by MHWs. Still, all realms had significant positive trends for at least two metrics (Table S3). Specifically, we found significant positive trends for all five metrics over 40 years in the Arctic, Temperate Northern Pacific, and Temperate South Africa realms. Significant change points for the same realms showed increases in MHW days (since 1998), number of events (since 1998), mean intensity (since 1997), maximum intensity (since 1997) and cumulative intensity (since 1998) (Figures S2–S6, Table S3). By contrast, the Southern Ocean had significant negative trends for the number of events and MHW days (since 2006), but significant positive trends for mean and maximum intensity (since 2005) **(**Figures S2–S5, Table S3). The Temperate Northern Atlantic, Temperate Australasia and Temperate South America showed significant positive trends, except for mean intensity (first 2 realms) and number of events. Additionally, The Tropical Atlantic, Eastern Indo-Pacific, Central Indo Pacific and Western Indo-Pacific showed significant negative trends for mean intensity of MHWs, but these regions also had significant increasing cumulative intensity and number of MHWs (Figures S2–S6, Table S3). Similarly, the Tropical Eastern Pacific had significant positive trends in cumulative intensity, number of MHW days and events, and (non-significant) negative trends in mean and maximum intensity. Our, re-analyses of trend lines after break point changes showed that the Arctic had a dramatic increase in number of MHW days (40.9 more days per decade per pixel after 2002) whereas the Western Indo-Pacific had the highest increase for number of events (1.8 more events per decade per pixel after 2004). For intensity metrics, the Southern Ocean showed the highest trend increase for mean (0.253 °C warmer per decade per pixel after 2005) and maximum (0.341 °C warmer per decade per pixel after 2005) intensity, whereas the Tropical Eastern Pacific had highest negative trend line in mean intensity (0.366 °C colder per decade per pixel after 2016 although this change point was not significant). Finally, the Temperate Northern Pacific showed the highest increase in cumulative intensity with 66.9 °C days more per decade per pixel after 2002.

### Seasonal trends within realms

**Figure 3:**
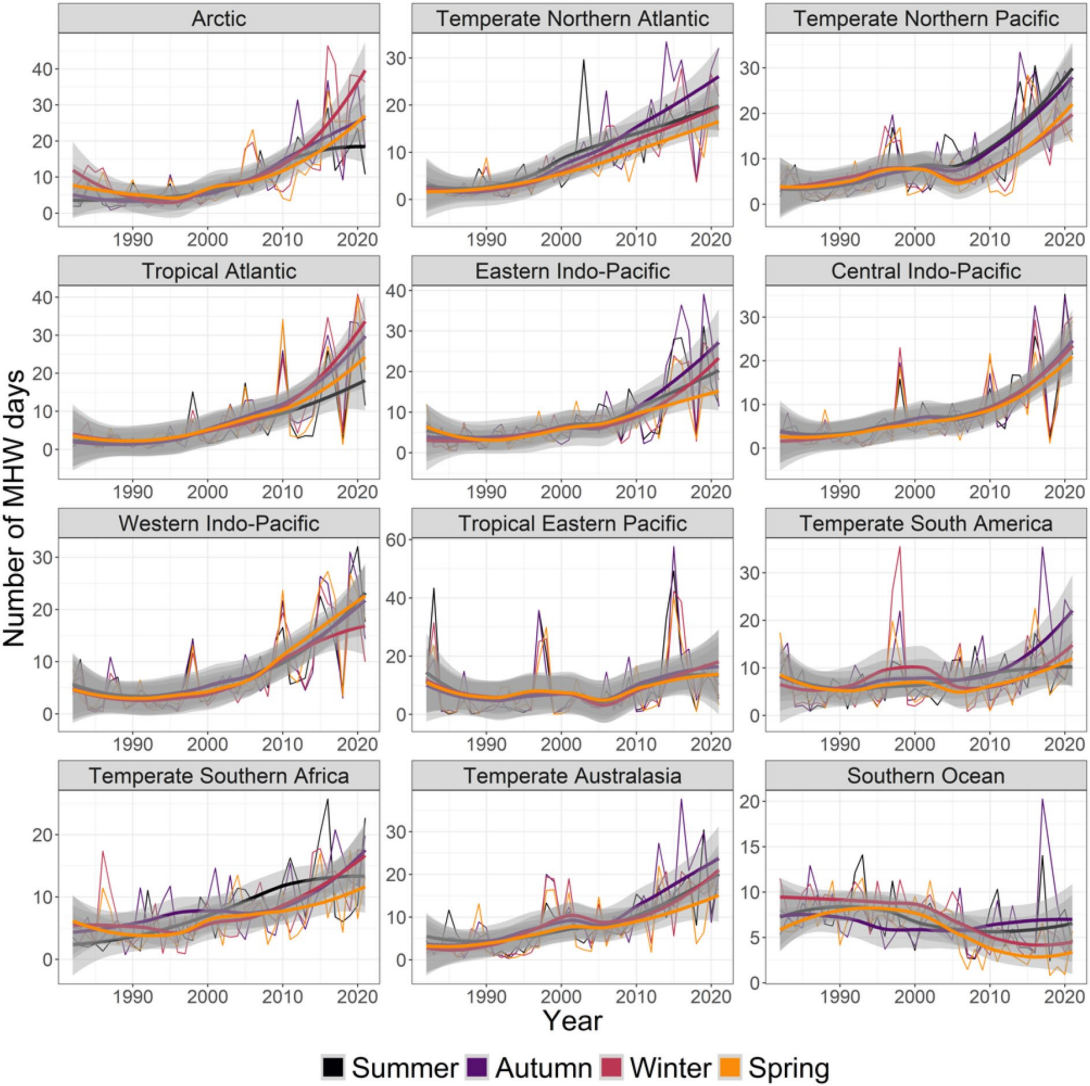
Seasonal trends from 1982 to 2021 in yearly- and pixel-averaged marine heatwave days per coastal realm and season. See Fig. 2 caption for more details and Table S4 for specific trendlines, *p* values and break point years.

**Figure 4:**
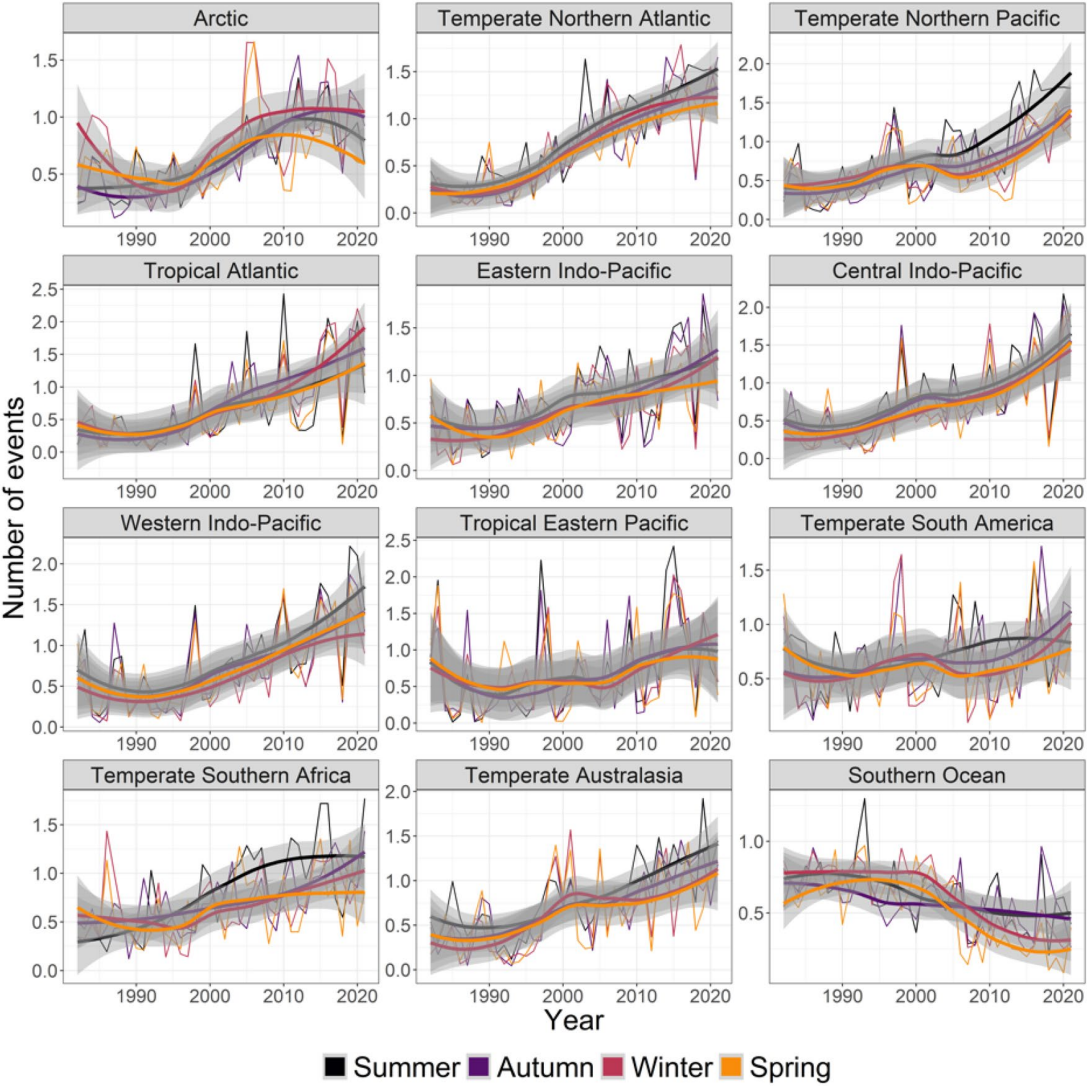
Seasonal trends from 1982 to 2021 in the yearly- and pixel-averaged number of marine heatwaves (events) per coastal realm and season. See Fig. 2 caption for more details and Table S4 for specific trendlines, *p* values and break point years.

Across realms, seasons, and metrics, 167 cases increased, 13 decreased, and 60 were non-significant (Fig. 1, Table S4). Most realms experienced significantly more MHWs per year and season: 48 cases for number of days and 39 cases for number of events, compared to regions and seasons with no changes (3 cases for days and 5 for events) or with fewer MHWs (3 cases for days and 4 for events) (Figs. 1, 3, 4, Table S4).

The Southern Ocean was the only region where the number of MHW days and number of events decreased, and this happened across summer, winter, and spring (MHW days) and all four seasons (number of events) with negative slopes ranging from 1.5 to 0.6 less MHW days per season per decade and change point years between 1993 and 2005. Realms without changes in MHW days included the Tropical Eastern Pacific (spring), and Temperate South America (spring). Of the 42 significant positive slopes, the five most dramatic increases (largest decadal trend shown in brackets, followed by change point year) were the Temperate Northern Atlantic in autumn (5.7 more days per decade, 1998), the Arctic in autumn (4.9, 2000), the Temperate Northern Atlantic in summer (4.9, 2001), the Temperate Northern Pacific in summer (4.8, 2003) and the Tropical Atlantic in autumn (4.6, 2001). However, slopes increased, and the rankings changed, in re-analysis after break change point, with largest changes for the Tropical Atlantic in winter (13.3, 2001) followed by the Arctic in winter (13.2, 2004), the Western Indo-Pacific in spring (11.3, 2004), the Eastern Indo-Pacific in autumn (10.6, 2001) and the Western Indo-Pacific in summer (10.5, 2004).

**Figure 5:**
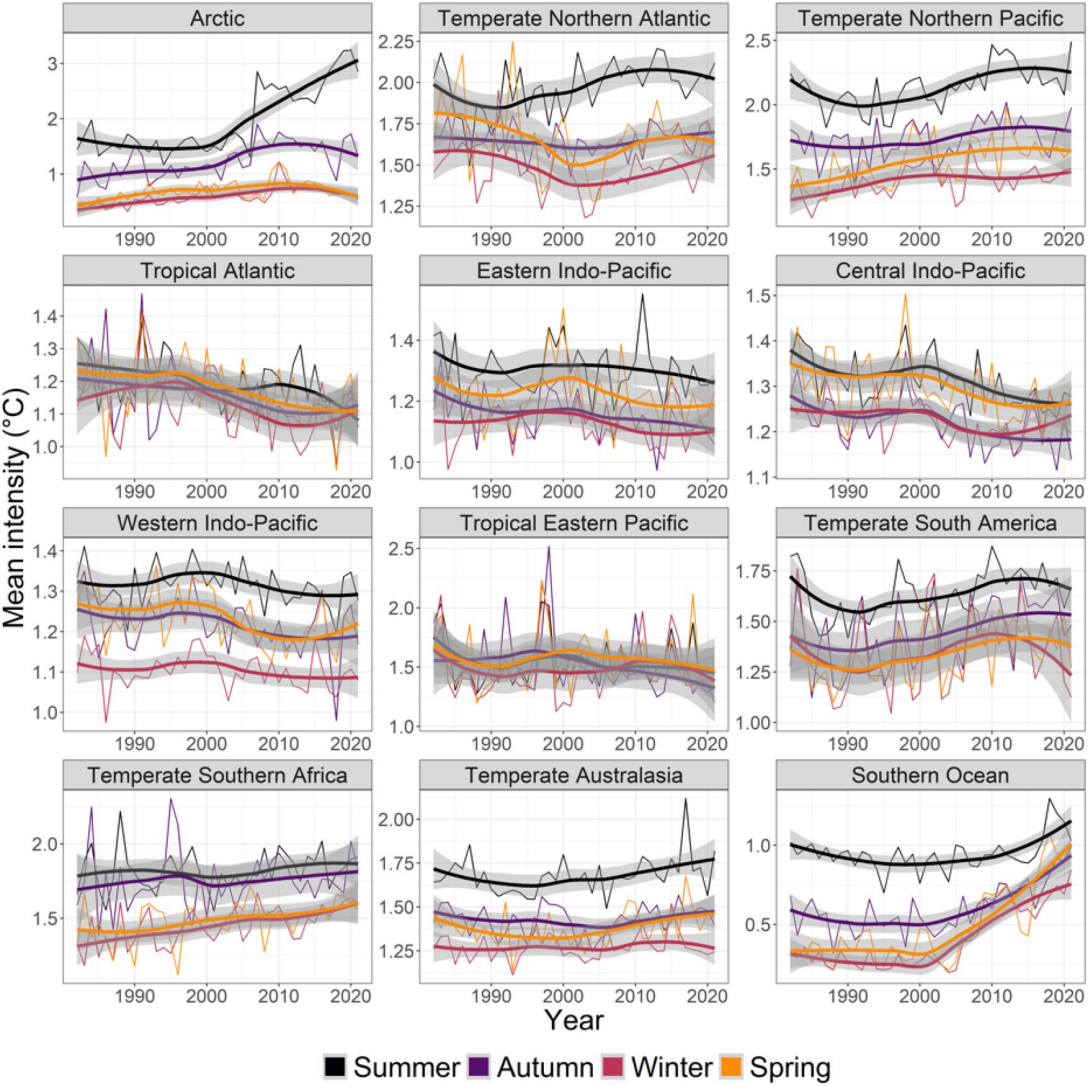
Seasonal trends from 1982 to 2021 in the yearly- and pixel-averaged mean intensity (°C) of marine heatwaves per coastal realm and season. See Fig. 2 caption for more details and Table S4 for specific trendlines, *p* values and break point years.

**Figure 6:**
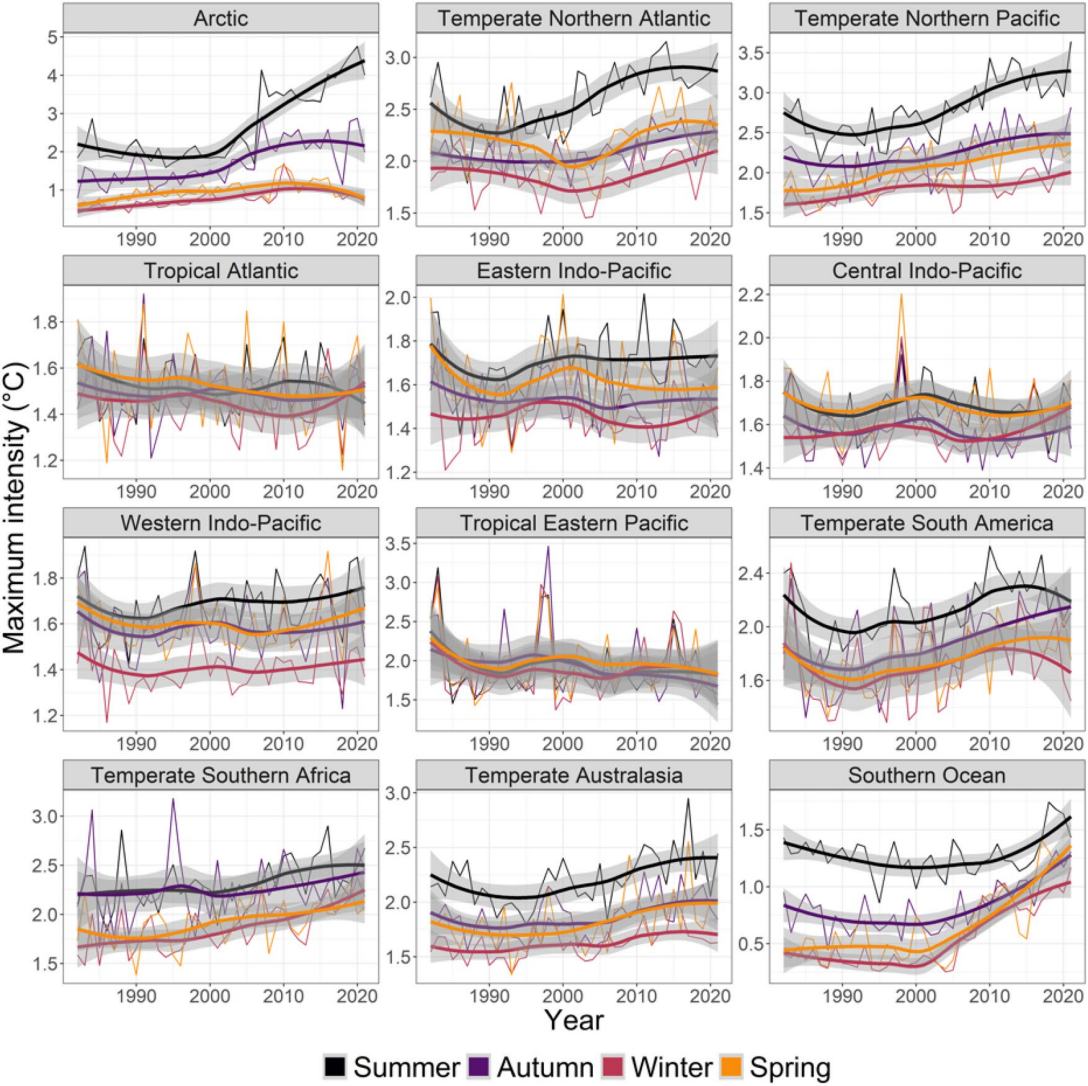
Seasonal trends from 1982 to 2021 in the yearly- and pixel-averaged maximum intensity (°C) of marine heatwaves per coastal realm and season. See Fig. 2 caption for more details and Table S4 for specific trendlines, *p* values and break point years.

Results for average MHW mean intensity were less clear, with 17 increasing, 21 non-significant changes, and 10 decreasing cases (Figs. 1, 5, Table S4). Realms with significant decreases included the Tropical Eastern Pacific (autumn), the Tropical Atlantic (spring, summer, winter), and the Central Indo-Pacific (winter) with negative slopes ranging from 0.027 to 0.046 °C less per decade and break change-year between 1996 and 2011). The strongest increases in mean MHW intensity were found in the Arctic in summer (0.359 °C more per decade, 2006) and autumn (0.191, 2002), the Southern Ocean in spring (0.143, 2005) and winter (0.111, 2005) and the Arctic in winter (0.086, 1998). By comparison, results for average MHW maximum intensity were clearer, with 22 increases, 26 non- significant effects, and 0 decrease (Figs. 1, 6, Table S4). The strongest increases in maximum MHW intensity were in the Arctic in summer (0.555 °C more per decade, 2006) and autumn (0.351, 2002), the Temperate Northern Pacific in summer (0.205, 2003), the Southern Ocean in spring (0.197, 2005) and the Temperate Northern Atlantic in summer (0.196, 2001).

**Figure 7:**
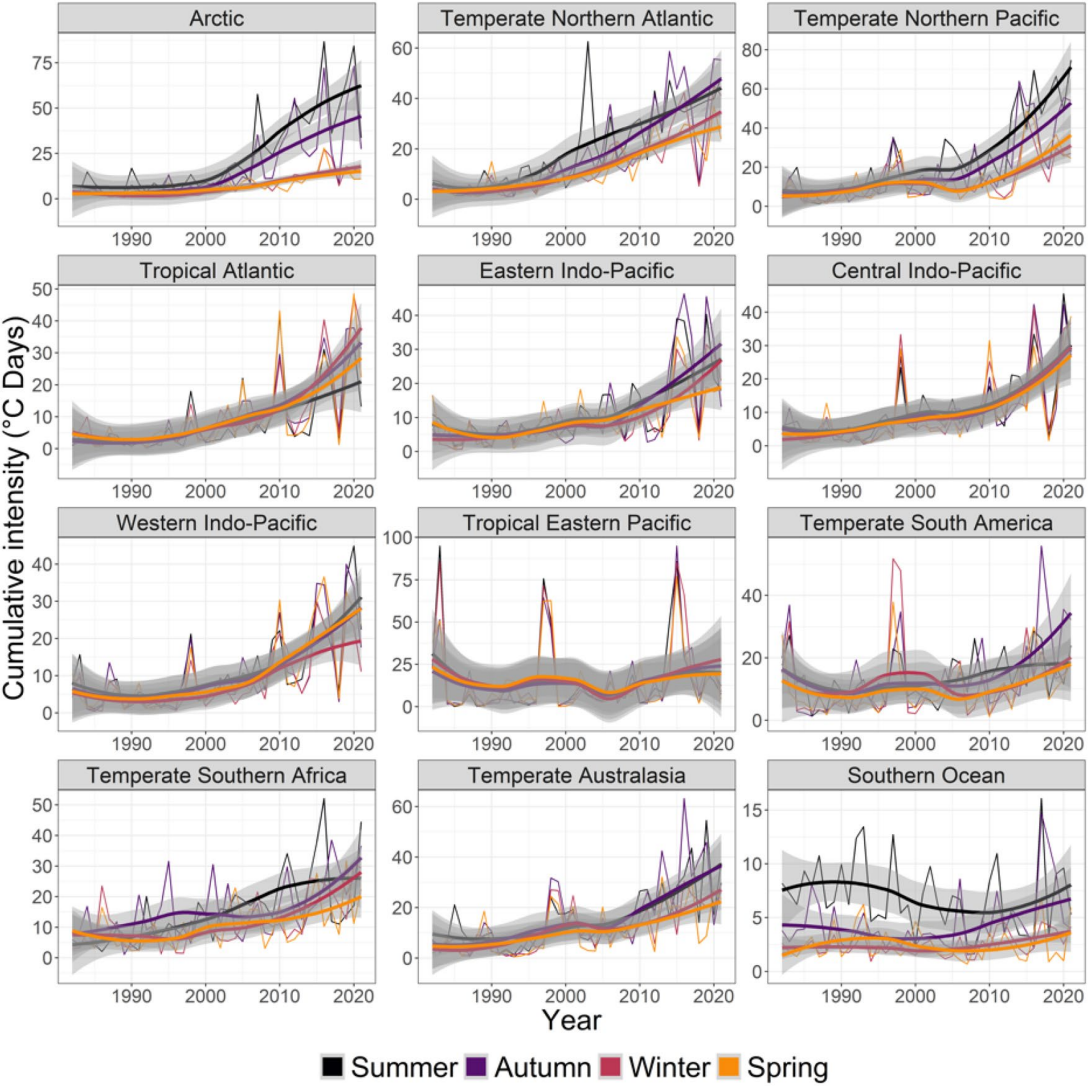
Seasonal trends from 1982 to 2021 in the yearly- and pixel-averaged cumulative intensity (°C days) of marine heatwaves per coastal realm and season. See Fig. 2 caption for more details and Table S4 for specific trendlines, *p* values and break point years.

Overall, MHW trends were strongest and clearest for cumulative intensity; 43 trends increased whereas 5 cases were non-significant (spring in Tropical Eastern Pacific and all seasons for the Southern Ocean, Figs. 1, 7, Table S4). Of the 43 increasing slopes, the strongest trends were observed for the Arctic in summer (12.0 °C days more per decade, 2002), the Temperate Northern Pacific in summer (11.2, 2003), the Temperate Northern Atlantic in summer (10.7, 2001) and autumn (9.8, 1998) and the Arctic in autumn (8.0, 2002). Generally, the realm × season ranking of the five steepest slopes, re-analysed after the break change year, were relatively similar although slopes increased by a factor of around 2 after the break change (1998–2003, ranging from 16.4 to 28.1 °C days more per decade).

In summary, across the 240 trend analyses, 70% increased significantly, 5% decreased and 25% were unaffected, highlighting that MHW metrics overall increased but with different magnitudes across metrics, realms, seasons, and time-periods. Finally, we found mostly similar results when the same set of analyses were applied to the most narrow coastline (i.e., a single coastline pixel), with 60% of cases increasing, 9% decreasing and 31% being non-significant, and generally with slightly steeper slopes (see Figures S7–S13).

## Discussion

Coastal ecosystems produce around half of earth’s ecosystem services^34^, but these valuable systems are stressed by land runoff, overfishing, invasive species, climate change^35,36^ and MHWs^2^. MHWs have had large impacts on many of the realms we analysed^1,2^, including loss of kelp in Temperate Australasia^8,17,20,37–39^, Northern Pacific^40^ and Northern Atlantic^41^, loss of seagrass in Temperate Australasia and the Central Indo-Pacific^18,42^, coral bleaching in Temperate Australasia, the Central Indo-Pacific and the Eastern Indo-Pacific^11,19,43,44^, invertebrate mass mortality in Temperate Australasia^12,45^, and fisheries closures and range shifts in Temperate Northern Pacific and Temperate Australasia^2,12,46,47^. Moreover, even though trends in MHW metrics were relatively weak for Temperate Australasia (cf. Figures 3, 4, 5, 6), extreme events in Western Australia (2010/11) and the South Island of New Zealand (2017/18) have had major ecological implications^8,17,20,24,37,38^. These case-studies highlight the importance of analysing MHWs in a coastal biogeographical context—where organisms share temperature adaptations—whereas regional events may be blurred on the oceanic scales used in many other analyses^4,5,15^.

Ecologically, the SST data analysed here in a MHW context are particularly relevant for epipelagic organisms, such as phytoplankton, many zooplankton, fish, and marine mammals^48–50^ as well as nearshore species, because coastal waters are often well-mixed. However, when and where strong stratification occurs, deep-water benthic organisms may experience different temperature conditions and therefore also different MHWs^48^. A mismatch between satellite-derived SST measurements and local benthic temperatures may therefore be problematic in deep offshore oceanic waters. Furthermore, matching satellite-derived SST to in situ temperature measured with loggers is challenging in the coastal zone^51–54^, suggesting that results from using all bioregional pixels are more robust than our analysis on the most coastal pixels (Figure S7–S13). Interestingly, Marin et al.^55^ found that trends in satellite-derived SST, number of MHW days and yearly cumulative intensity of MHWs appeared generally weaker in the coastal zone compared to offshore pixels suggesting a potential role as thermal refugia for the coastal zone, like shown for upwelling areas^56,57^. Still, there were regional disparities, and mean MHW metrics were generally higher in the coastal zone, including upwelling regions, highlighting that differences in onshore-offshore trends can be complex and depend on the analysed metrics.

Biological impacts have been evaluated from many warm-water episodes in the past, such as after El Niño events in 1982/83, 86/87, 91/92 and 97/98^58–62^. However, these analyses correlated impacts to temperature or temperature anomalies, rather than MHW metrics. Furthermore, many coral reef studies have evaluated temperature impacts with a coral degree-heating-week index^63,64^ targeting a specific taxonomic group without clear linkages either to the maximum intensity or duration of warm events. El Niño events oscillate on long time scales and are therefore relatively rare in our time-series, and their contribution to trends and change point detection therefore remain limited. Nevertheless, graphical analysis shows large spikes in several MHW metrics around these events (Figs. S3–S7), with some events being superimposed on increasing trendlines (e.g., cumulative intensity and MHW days in the Tropical Eastern Pacific and Temperature South America realms, Figs. 3, 7). We suggest that re-analysis of past warm-water events, like El Niño, with MHW metrics and underlying trendlines may increase the understanding of the mechanisms causing detrimental ecological temperature effects^58–62^. Complementary analyses of MHW metrics should provide better understanding of drivers of biological changes compared to analysis of a single metric. For example, regions with increasing cumulative intensity could be driven by increasing MHW days and number of events (e.g., Central Indo-Pacific, Tropical Atlantic), by an increase in intensity and duration metrics (like temperate realms and the Arctic) or by contrasting trends in MHW days versus mean and maximum intensity (like in the Southern Ocean) (Figs. 1, 3, 4, 5, 6, 7). Few studies have compared metrics on different biological responses; for example, Smale et al.^1^ found strong negative correlations between MHW days and coral bleaching, seagrass density and kelp cover but less clear correlations with maximum intensity, suggesting that these foundation species responded to the length of temperature stress. By contrast, Montie et al.^13^ found a strong correlation between chlorophyll-a concentration (a proxy for phytoplankton abundance) and maximum intensity of summer MHWs, but not their duration, perhaps because phytoplankton have extremely short turnover times and can respond rapidly to short but strong events.

Most MHW impact studies have focused on summer events^1,9,13,17,18,20,65^, where effects likely are greatest, because species living near their distributional range limit experience ambient temperature close to their upper thermal limits^66,67^. In these cases, elevated summer temperatures can push foundation species like corals, seagrass, and kelp beyond their thermal tolerance, with immediate consequences^40,54^. However, all ecological processes, like survival, growth, reproduction, and species-interactions change throughout seasons^26^. A MHW at any given time may therefore alter normal seasonal patterns by exceeding species-specific thresholds, affect growth and productivity, stimulate earlier ontogenetic onsets, increase respiration and decomposition, or alter predation rates and competitive hierarchies^27,68–70^. For example, we found that maximum and cumulative MHW intensities increased in Arctic summer (Fig. 6, 7) when light levels are highest, potentially facilitating phytoplankton growth and reproduction^13,71^. However, MHWs are also getting longer in darker winter months (Fig. 3) so that low light levels may impede phytoplankton responses. Still, for ectotherms like fish and benthic invertebrates, growth, feeding and reproduction could be affected by longer Arctic winter MHWs^72^. The ecological importance of seasonality in MHWs is expected to peak at mid to high latitudes and be weakest near the equator (with less seasonality)^73,74^. Future analysis of seasonal changes to MHW in tropical regions could re-focus on wet-dry seasons, monsoons, or specific organisms’ life-histories, instead of the three-month-interval used here. Follow-up studies could also use retrospect analysis to test if the seasonal changes to tropical MHWs found here (e.g., strong increases in cumulative intensity of MHWs in Tropical Atlantic, Eastern Indo-Pacific, and Central Indo-Pacific) have affected local communities.

Identifying physical drivers of MHWs is an active research field^15^ of fundamental importance to developing forecasting tools^75^, like predicting onsets and declines of MHWs^76^. MHWs in surface waters can be driven by advection, increased air-sea fluxes and reduced vertical mixing which are ultimately induced by complex climate processes^15^. Our analytical approach could be applied to specific coastal regions defined by coherent water masses, such as eastern and western boundary currents and their extensions, or climate modes^15^ as well as finer bioregional scales than realms such as within provinces or bioregions. Similarly, change point analyses from long-term SST satellite data could help identify periods when underpinning physical drivers (atmospheric or oceanographic) have changed.

Here we expanded on three key coastal studies^24,55,77^ adding detailed analyses of biogeographical and seasonal effects with new robust linear regressions and break point detections. We conclude that coastal MHWs, globally, are becoming more frequent, longer, and stronger, but also that these trends conceal high variability between seasons and biogeographical realms, in both direction (positive vs. negative slopes) and magnitude (small-to-large). We also documented, from break point analyses, general acceleration in MHW metrics, across seasons and bioregions. A recent global meta-analysis of ecological impacts, and new case studies, have largely analysed impacts from summer MHWs from a few realms^1,2^. Little is therefore known about MHW impacts for many other realms, and particularly how stronger and longer MHWs in winter, spring, or autumn have affected coastal ecosystems. We also highlight that some coastal regions that have experienced strong increases in MHWs are very much under-sampled, including the Arctic region and remote temperate and equatorial regions. If the trends we detected continue, there will be a need for greater biogeographical coverage of reporting ecological impacts from MHWs in these regions.

## Methods

### Coastal Sea surface temperature and detection of marine heatwaves

Daily Optimum Interpolation Sea Surface Temperature (OISST v2.1) provided by the United States National Oceanic and Atmospheric Administration (NOAA) was downloaded for all pixels covering the realms of the worlds marine ecoregions out to 200 nautical miles (370 km)^22^ (n = 260,010 pixels). The OISST v2.1 dataset has been constructed combining observations from satellites, ships, buoys and Argo floats, and interpolated to a 1/4° degree regular grid, allowing continuous estimates of sea surface temperature in space and time^78^. We downloaded and analysed daily SST values between 1 January 1982 and 31 December 2021. To investigate if coastal patterns in MHW trends varied with distance from the coastline, we ran the same analysis on all 260,010 coastal pixels as well as using a single most coastal pixel band (n = 16,160 pixels), where the former represents the previous described bioregions, and the latter the area inhabited by coastal foundation species (that only live in a narrow coastal band) where they may experience wider temperature fluctuations. To select the most coastal pixels, we used the Distance to Nearest Coastline at 0.01 Degree Grid dataset provided by NOAA^79^ and created a coastal mask from the coastline to 10 km offshore. Every OISST pixel intersecting the first 10 km-from-the-coastline band was kept for the analysis. Following this masking procedure, only the most coastal 1/4° degree pixels were kept along the coastline, corresponding to 16,160 pixels. However, the narrow coastline pixels may be prone to errors^51–54^, so here we focus on results from the coastal biogeographical realms and only show results from the narrow coastline band in the online supplement for comparisons. From the daily OISST coastal time series, MHWs were detected with the “heatwaveR” R package (version v0.4.6)^80^. The 30-year climatology period from 1 January 1983 to 31 December 2012 was used to calculate the SST mean and daily MHW intensity, which again was used to quantify five key MHW metrics: (a) the number of MHW days (in days), (b) the number of events, (c) the mean intensity (the mean temperature anomaly measured relative to the climatological, seasonally-varying mean, in °C), (d) the maximum intensity (the maximum temperature anomaly measured relative to the climatological, seasonally-varying mean, in °C) and (e) the cumulative intensity (integrated temperature anomaly over the season/year, in °C days). These analyses were run for every pixel OISST time series within the coastal realms, giving an output of the history of MHWs per individual pixel since January 1982. The metrics were then summed or averaged per year, season and/or realms. Here we analysed NOAA OISST data only, although SST data derived from other satellite products are possible. Nevertheless, we used the global 40-year NOAA OISST product because it is longest and therefore increases the likelihood of detecting trends and temporal change points and is the most used product. However, The Japan Meteorological Agency (JMA) have merged satellite and in situ global daily SST^81^ and these data could also be used in future analysis because they cover similar spatial and temporal resolution. Most importantly, Marin et al.^24^ reported relatively consistent MHW results between different SST satellite products, only with some minor differences between MHW mean intensities (i.e., our intensity results should therefore be interpreted slightly more cautiously).

### Trend and change-point detection across global, regional, and seasonal scales

To detect temporal trends in MHW metrics, we summarised daily MHW metrics by year and season. Seasons were classified as traditional 3-month time windows with opposite months for the northern (> 0°N) and southern (< 0°N) hemispheres. In the northern hemisphere June–August was summer, September–November was autumn, December–February was winter, and March–May was spring. Mann–Kendall, Theil–Sen (Sen’s slope hereafter) and Pettit change points were calculated using The R package “trend” (version 1.1.4)^33^ on the time series for each MHW metric. Here, Mann–Kendall trend tests are non-parametric, distribution-free, and often used in environmental and climate studies^32,33^. The non-parametric Sen’s linear slopes estimate the direction and intensity of trends, a method that is robust to outliers compared to standard parametric linear regression^32,33^. Finally, non-parametric Pettitt change point tests detect shifts in central tendency, which would indicate significant changes in the temporal dynamics. Pettitt *p* values can, in a few cases, exceed one^33^ but are listed as 1 in the online tables. We subsequently used the change point year to re-calculate trends before and after this change point year. Finally, decadal trends were tested on the 30-year time series and on time series before and after change points, for each individual MHW metric by combining pixels across (a) the entire world, (b) for each of the 12 coastal realms, (c) for each of the four seasons, and (d) for each combination of 12 coastal realm and 4 seasons. Some MHW metrics scale with the number of pixels that characterize a bioregion and we therefore divided the number of MHW days, number of events and cumulative intensity by the number of pixels per realm (i.e., these pixel-averaged yearly values are also directly comparable between realms). Scaling metrics for realms with different number of pixels as well as different pixel areas (latitudinal dependent) does not appear to be a caveat of our study conclusions as we found similar patterns between using different pixels (all pixels within realms vs most coastal).

## Supplementary Information

Supplementary Information.

## Data Availability

NOAA OISST v2.1 data are freely available at https://www.ncei.noaa.gov/products/optimum-interpolation-sst (last accessed 28/02/2022). The R package used to calculate MHW metrics, “heatwaveR”, is available on CRAN and https://robwschlegel.github.io/heatwaveR/ (version v0.4.6, last accessed 28/02/2022), with instructions for how to download NOAA OISST data. A web-based app summarising the global, seasonal, and bioregional trends is freely accessible at https://frantoto.shinyapps.io/Global_Coastal_Seasonal_Trends_MHW_Metrics/ (last accessed 28/02/2022). The code related to this analysis is available at https://github.com/FranToto/Thoral2021_GlobalCoastalSeasonalMHW and https://rpubs.com/FranToto/GlobalMHWTrends (last accessed 28/02/2022).
