# Supplementary material for: Unravelling seasonal trends in coastal marine heatwave metrics across global biogeographical realms
Source: Sci Rep. 2022 May 11;12:7740. doi: 10.1038/s41598-022-11908-z (PMC9095592; doi:10.1038/s41598-022-11908-z)
Supplement: Supplementary file 1 — Supplementary Information. [file 41598_2022_11908_MOESM1_ESM.docx]

## Supplementary Information

**Figure S1.** Seasonal trends from January 1982 to December 2021 in the number of yearly-averaged and pixel-averaged marine heatwave days, number of events, mean intensity (°C), maximum intensity (°C) and cumulative intensity (°C days) of marine heatwaves in coastal realms (thin lines). The thick lines show loess smoothing (+ the 95% confidence interval around smoothing), indicating potential breakpoint in linear trend analysis.

**
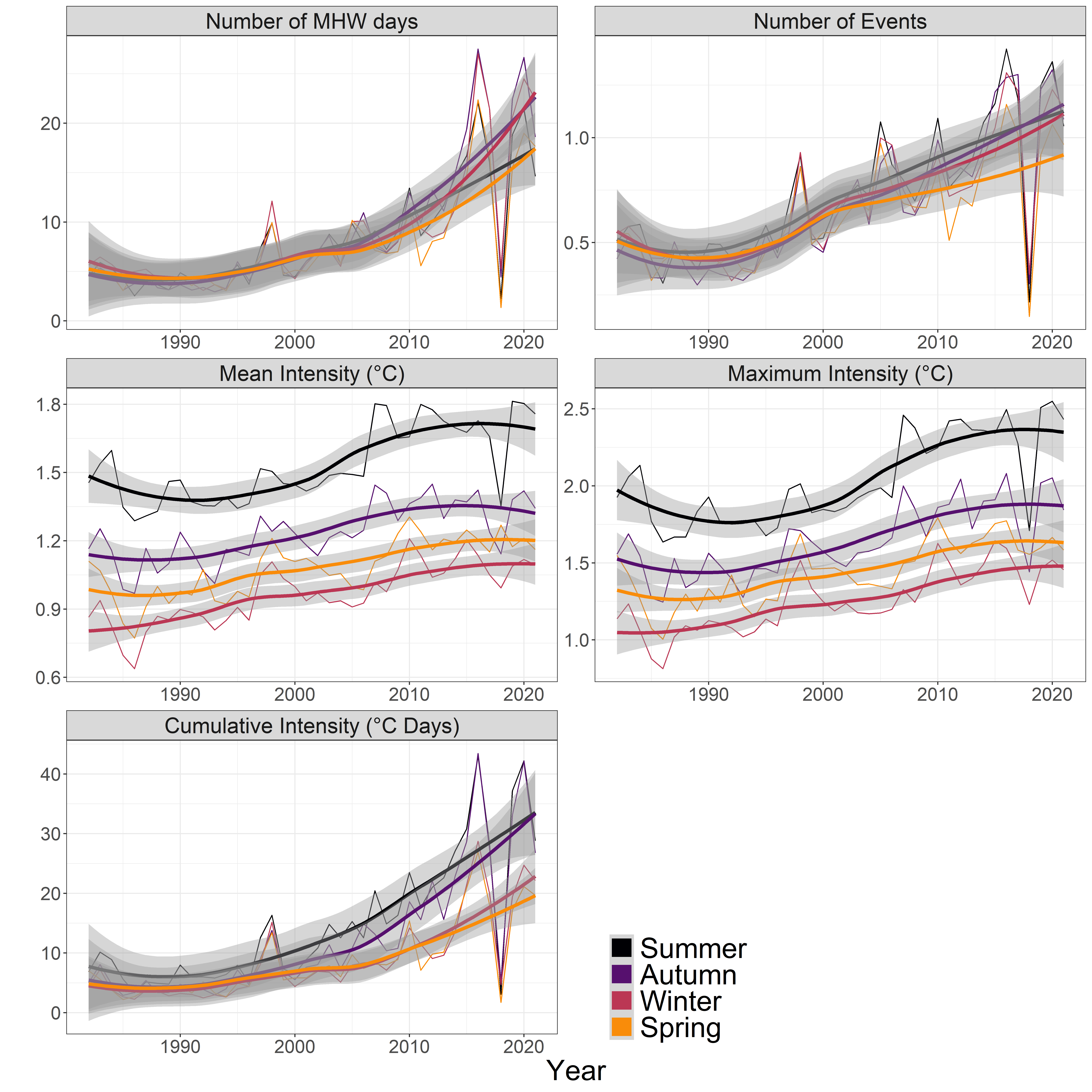
**

**Figure S2.** Yearly trends from 1982 to 2021 in the yearly-averaged and pixel-averaged marine heatwave days per coastal realm. See caption of figure S1 for similarities in figure legend.


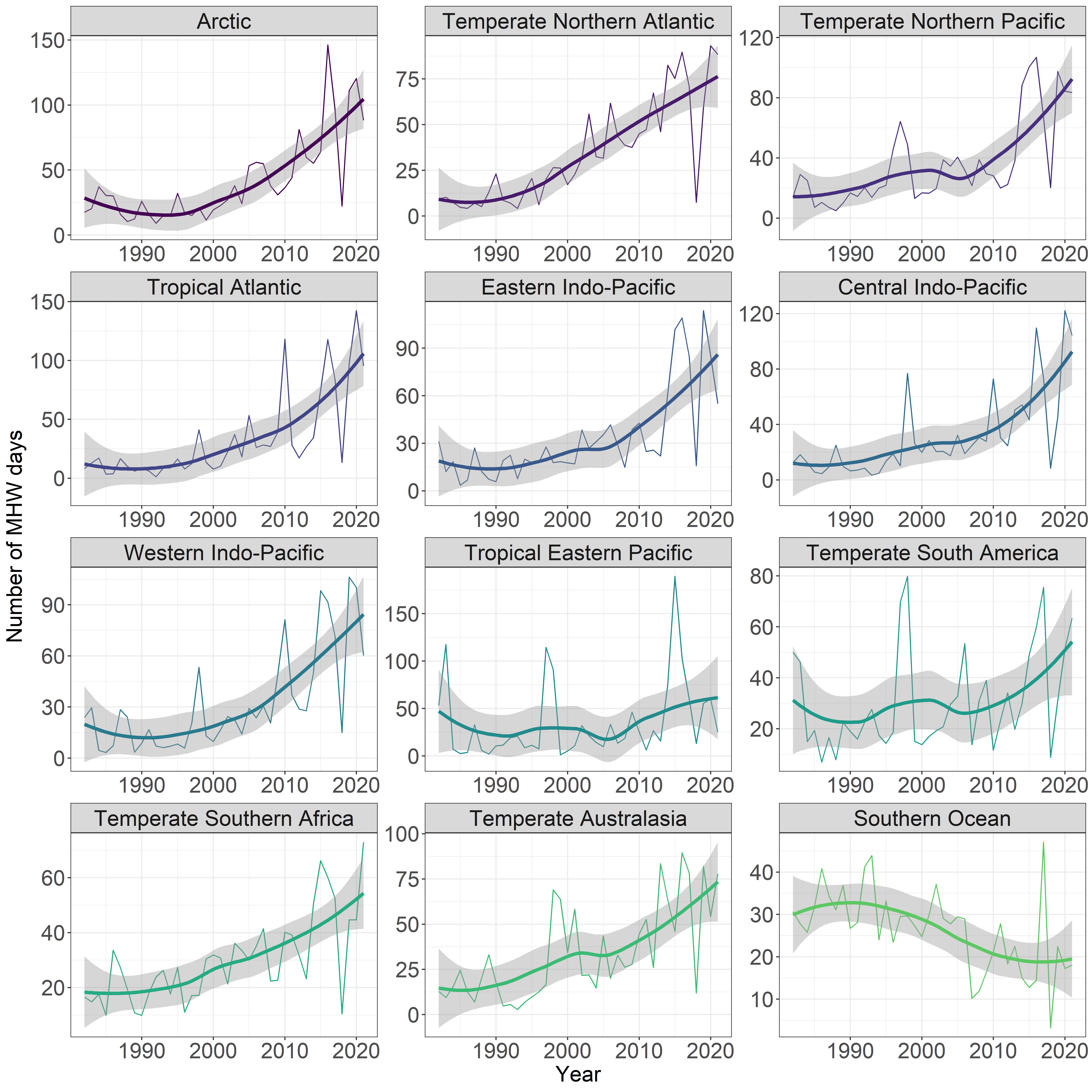


**Figure S3.** Yearly trends from 1982 to 2021 in the yearly-averaged and pixel-averaged number of marine heatwaves (events) per coastal realm. See caption of figure S1 for similarities in figure legend.


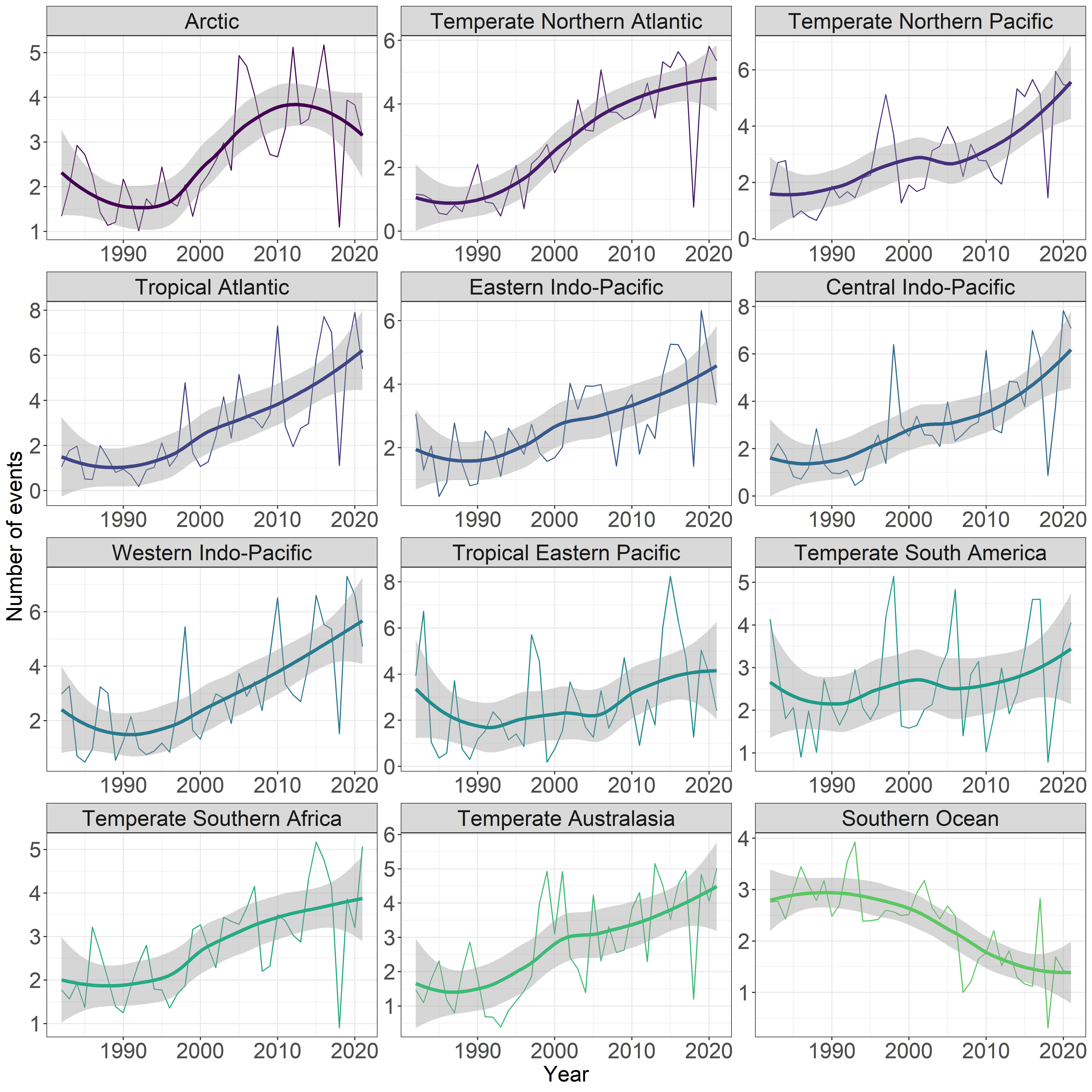


**Figure S4.** Yearly trends from 1982 to 2021 in the yearly-averaged and pixel-averaged mean intensity (°C) of marine heatwaves per coastal realm. See caption of figure S1 for similarities in figure legend.


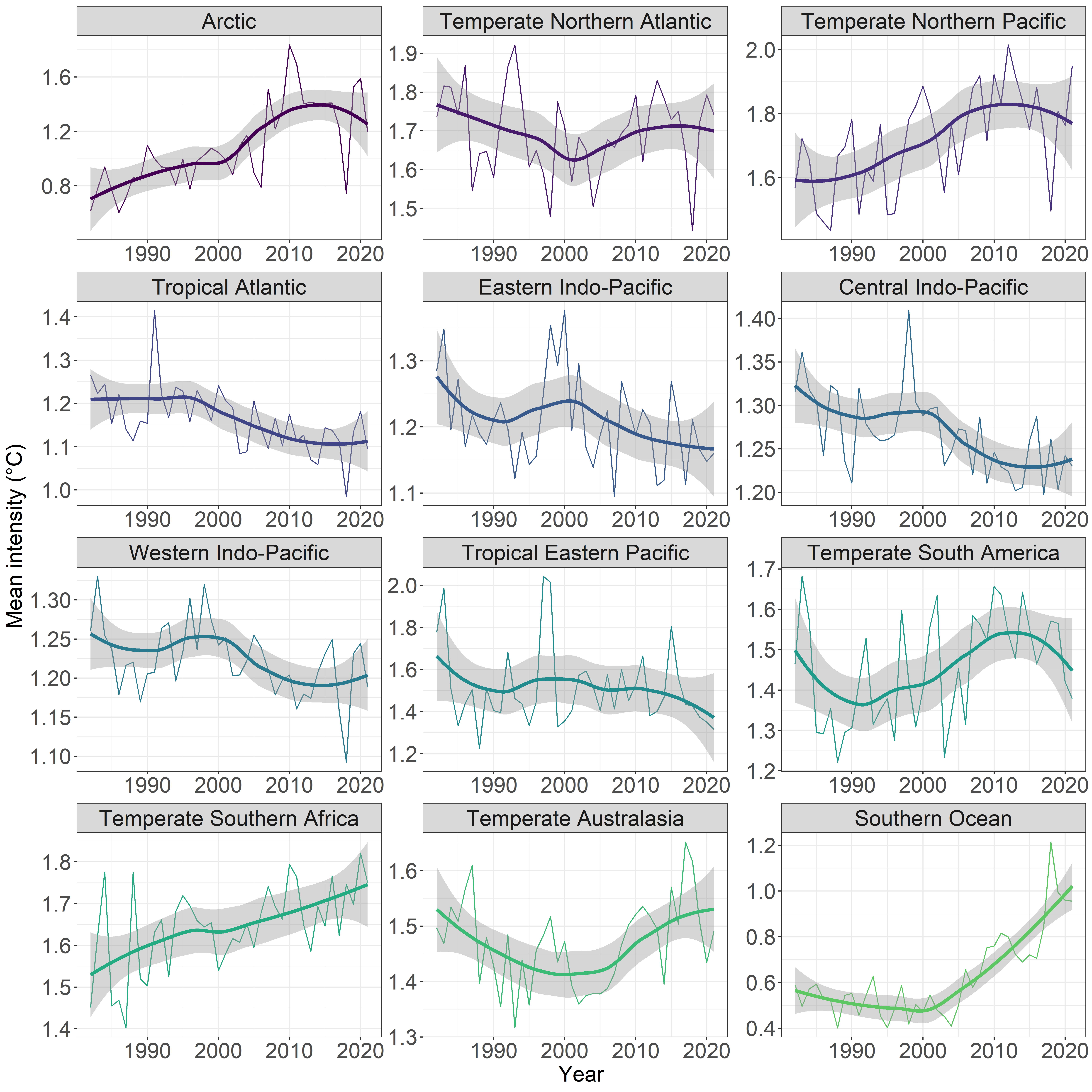


**Figure S5.** Yearly trends from 1982 to 2021 in the yearly-averaged and pixel-averaged maximum intensity (°C) of marine heatwaves per coastal realm. See caption of figure S1 for similarities in figure legend.


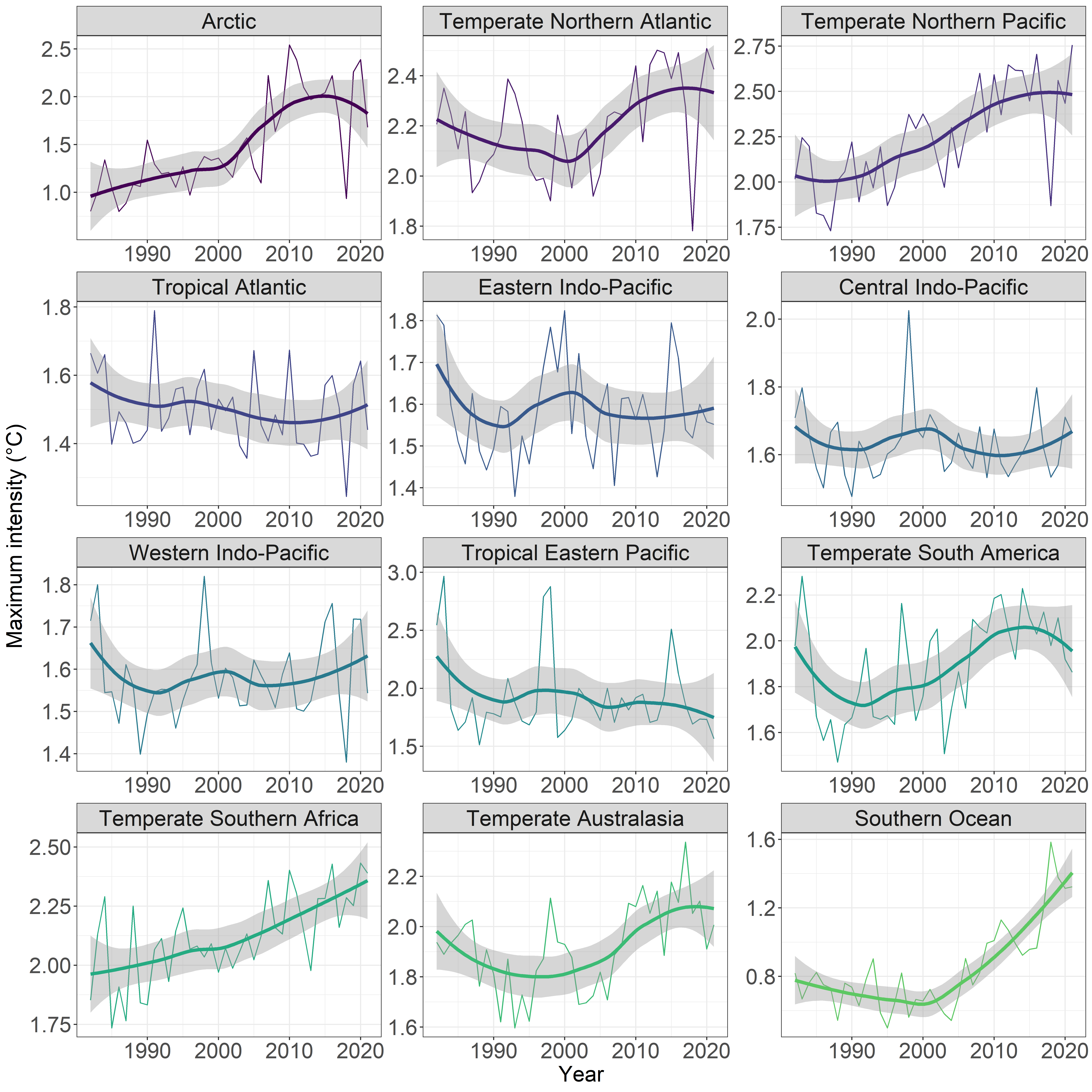


**Figure S6.** Yearly trends from 1982 to 2021 in the yearly-averaged and pixel-averaged cumulative intensity (°C days) of marine heatwaves per coastal realm. See caption of figure S1 for similarities in figure legend.


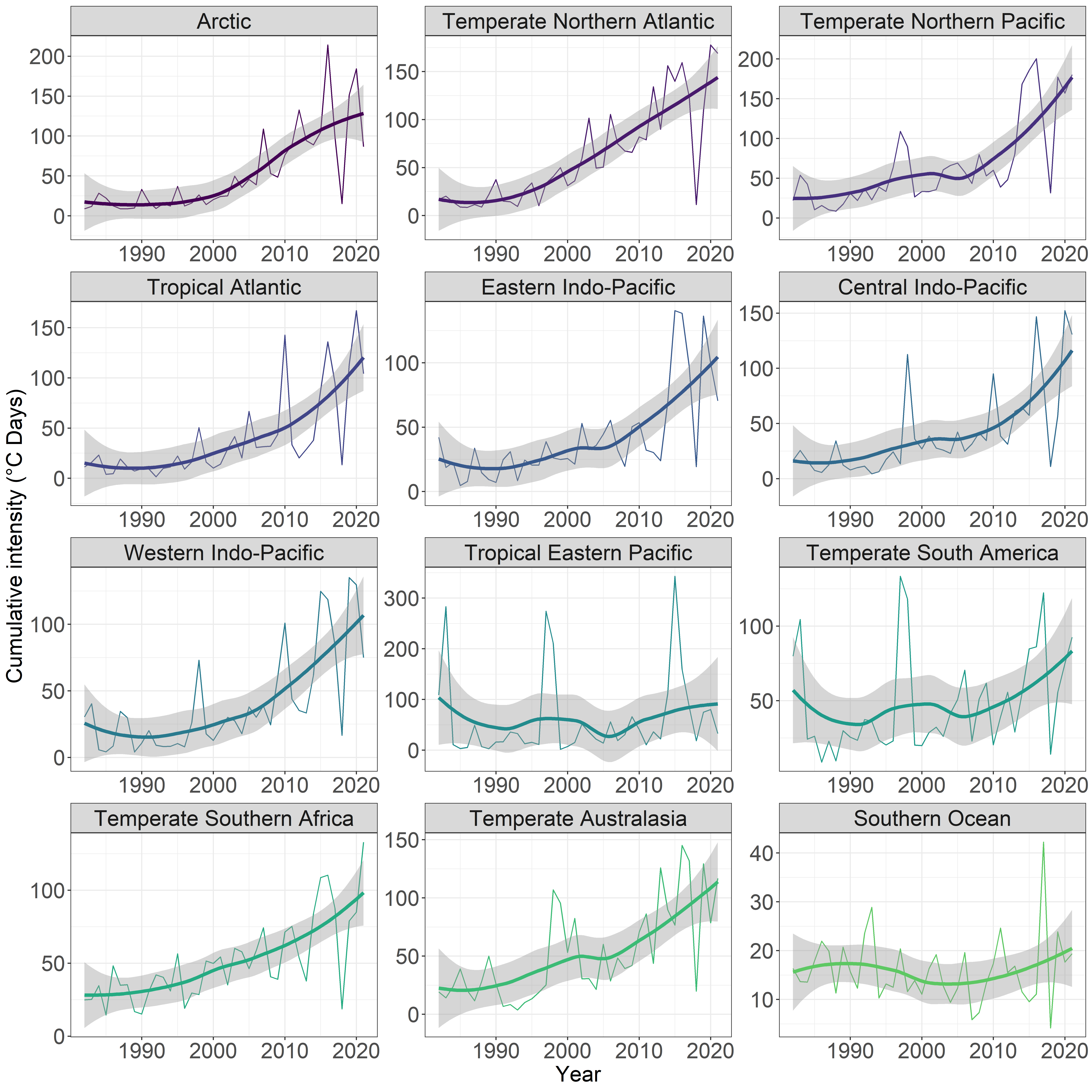


| **Table S1.** Decadal trends in global yearly MHW Metrics (Sen’s Slope*10, Mann-Kendall p-value, and change-point detection) between 1982 and 2021. Decadal trends pre and post break point year and their ratio are also shown. | | | | | |
| --- | --- | --- | --- | --- | --- |
| Metric | Decadal Trend  (p-value) | Year Change Point  (p-value) | Trend Pre-Change Point  (p-value) | Trend Post-Change Point  (p-value) | Ratio Trends Post/Pre-Change Point |
| MHW days | 11.698  **(< 0.0001)** | 2001  **(< 0.0001)** | 1.388  (0.581) | 28.874  **(0.0001)** | 20.8 |
| Events | .744  **(< 0.0001)** | 2001  **(< 0.0001)** | 0.211  (0.144) | 0.994  **(0.003)** | 4.7 |
| Mean intensity | 0.088  **(< 0.0001)** | 1996  **(< 0.0001)** | -0.008  (0.767) | 0.079  **(0.006)** | 9.9 |
| Max. intensity | 0.154  **(< 0.0001)** | 2004  **(1)** | 0.084  (0.113) | 0.163  **(0.034)** | 1.9 |
| Cum. intensity | 19.720  **(< 0.0001)** | 2001  **(< 0.0001)** | 5.546  (0.086) | 40.547  **(< 0.0001)** | 7.3 |

| **Table S2.** Trend in Global MHW Metrics across Season (Sen’s Slope*10 and Mann-Kendall p-value, and change-point detection). | | | | | | |
| --- | --- | --- | --- | --- | --- | --- |
| Season | Metric | Decadal Trend  (p-value) | Year Change Point  (p-value) | Trend Pre-Change Point  (p-value) | Trend Post-Change Point  (p-value) | Ratio Trends Post/Pre-Change Point |
| Summer | MHW days | 3.150  (**< 0.0001**) | 2002  **(< 0.0001)** | 0.846  (0.066) | 6.177  **(0.0007)** | 7.3 |
|  | Events | 0.203  (**< 0.0001**) | 2001  **(< 0.0001)** | 0.073  (0.074) | 0.279  **(0.002)** | 3.8 |
|  | Mean intensity | 0.112  (**< 0.0001**) | 2003  (**< 0.0001**) | 0.041  (0.430) | 0.099  (0.108) | 2.4 |
|  | Max. intensity | 0.195  (**< 0.0001**) | 2003  (**< 0.0001**) | 0.044  (0.499) | 0.253  **(0.021)** | 5.7 |
|  | Cum. intensity | 7.034  (**< 0.0001**) | 2002  (**< 0.0001**) | 1.704  (0.085) | 13.791  **(0.0001)** | 8.1 |
| Autumn | MHW days | 3.715  (**< 0.0001**) | 2001  (**< 0.0001**) | 0.630  (0.230) | 8.913  **(0.0003)** | 14.1 |
|  | Events | 0.224  (**< 0.0001**) | 2000  (**< 0.0001**) | 0.052  (0.294) | 0.335  **(0.0003)** | 6.4 |
|  | Mean intensity | 0.083  (**< 0.0001**) | 2005  (**< 0.0001**) | 0.049  (0.097) | 0.012  (0.837) | 0.2 |
|  | Max. intensity | 0.152  (**< 0.0001**) | 2004  (**< 0.0001**) | 0.070  (0.139) | 0.152  **(0.041)** | 2.2 |
|  | Cum. intensity | 5.901  (**< 0.0001**) | 2002  (**< 0.0001**) | 1.646  (0.037) | 13.681  **(0.0004)** | 8.3 |
| Winter | MHW days | 2.656  (**< 0.0001**) | 2004  (**< 0.0001**) | 0.525  (0.342) | 9.488  **(0.0040)** | 18.1 |
|  | Events | 0.192  (**< 0.0001**) | 2001  (**< 0.0001**) | 0.022  (0.626) | 0.248  **(0.0041)** | 11.4 |
|  | Mean intensity | 0.085  (**< 0.0001**) | 1996  (**< 0.0001**) | 0.023  (0.621) | 0.069  **(0.0136)** | 3.0 |
|  | Max. intensity | 0.135  (**< 0.0001**) | 1996  (**< 0.0001**) | 0.010  (0.843) | 0.124  **(0.0063)** | 12.7 |
|  | Cum. intensity | 3.482  (**< 0.0001**) | 1996  (**< 0.0001**) | -0.902  (0.428) | 5.846  (**< 0.0001**) | 6.5 |
| Spring | MHW days | 2.275  (**< 0.0001**) | 2001  (**< 0.0001**) | 0.240  (0.381) | 5.890  **(0.0015)** | 24.5 |
|  | Events | 0.148  (**< 0.0001**) | 1996  (**< 0.0001**) | -0.060  (0.235) | 0.173  **(0.0008)** | 2.9 |
|  | Mean intensity | 0.082  (**< 0.0001**) | 2006  (**< 0.0001**) | 0.045  (0.088) | 0.037  (0.3444) | 0.8 |
|  | Max. intensity | 0.130  (**< 0.0001**) | 2006  (**< 0.0001**) | 0.083  (0.107) | 0.077  (0.3004) | 0.9 |
|  | Cum. intensity | 3.205  (**< 0.0001**) | 1996  (**< 0.0001**) | -0.508  (0.767) | 4.915  **(0.0001)** | 9.7 |

| **Table S3.** Trend of MHW Metrics in across global coastal realms (Sen’s Slope*10 and Mann-Kendall p-value, and change-point detection). | | | | | | |
| --- | --- | --- | --- | --- | --- | --- |
| Realm | Metric | Decadal Trend  (p-value) | Year Change Point  (p-value) | Trend Pre-Change Point  (p-value) | Trend Post-Change Point  (p-value) | Ratio Trends Post/Pre-Change Point |
| Arctic | MHW days | 16.904  (**< 0.0001**) | 2002  (**< 0.0001**) | -1.312  (0.740) | 40.944  **(0.003)** | 31.2 |
|  | Events | 0.690  (**< 0.0001**) | 2001  (**< 0.0001**) | -0.035  (0.974) | 0.490  (0.174) | 14 |
|  | Mean intensity | 0.200  (**< 0.0001**) | 2006  (**< 0.0001**) | 0.133  **(0.010)** | 0.004  (0.964) | 0.03 |
|  | Max. intensity | 0.329  (**< 0.0001**) | 2006  (**< 0.0001**) | 0.179  **(0.010)** | 0.078  (0.822) | 0.4 |
|  | Cum. intensity | 27.549  (**< 0.0001**) | 2002  (**< 0.0001**) | 5.012  **(0.050)** | 57.778  **(0.004)** | 11.5 |
| Temperate Northern Atlantic | MHW days | 18.894  (**< 0.0001**) | 2001  (**< 0.0001**) | 8.412  **(0.025)** | 24.351  **(0.001)** | 2.9 |
|  | Events | 1.342  (**< 0.0001**) | 2001  (**< 0.0001**) | 0.771  **(0.021)** | 1.345  **(0.002)** | 1.7 |
|  | Mean intensity | 0.0001  (0.991) | 1994  (0.524) | 0.046  (0.669) | 0.045  (0.055) | .98 |
|  | Max. intensity | 0.076  **(0.006)** | 2005  **(0.003)** | -0.083  (0.097) | 0.136  **(0.044)** | 1.6 |
|  | Cum. intensity | 34.519  (**< 0.0001**) | 2001  (**< 0.0001**) | 13.577  **(0.010)** | 48.149  **(0.001)** | 3.5 |
| Temperate Northern Pacific | MHW days | 13.161  (**< 0.0001**) | 2002  (**< 0.0001**) | 6.224  (0.110) | 31.100  (0.064) | 5 |
|  | Events | 0.903  (**< 0.0001**) | 2002  **(0.001)** | 0.591  (0.085) | 1.420  **(0.041)** | 2.4 |
|  | Mean intensity | 0.078  (**0.0002**) | 1997  (**0.0006**) | -0.002  (1) | 0.033  (0.315) | 18 |
|  | Max. intensity | 0.178  (**< 0.0001**) | 1997  (**0.0002**) | 0.005  (1) | 0.160  **(0.013)** | 35.6 |
|  | Cum. intensity | 26.720  (**< 0.0001**) | 2002  (**0.0004**) | 13.474  (0.057) | 66.877  **(0.018)** | 5 |
| Tropical Atlantic | MHW days | 13.342  (**< 0.0001**) | 2001  (**< 0.0001**) | 1.926  (0.417) | 37.954  **(0.010)** | 19.7 |
|  | Events | 1.192  (**< 0.0001**) | 2001  (**< 0.0001**) | 0.284  (0.315) | 1.638  **(0.050)** | 5.8 |
|  | Mean intensity | -0.033  (**< 0.0002**) | 2002  (**0.0002**) | 0.001  (0.976) | -0.020  (0.496) | 24.2 |
|  | Max. intensity | -0.018  (0.357) | 2002  (0.730) | 0.008  (0.880) | 0.012  (0.721) | 1.6 |
|  | Cum. intensity | 15.004  (**< 0.0001**) | 2001  (**< 0.0001**) | 3.094  (0.417) | 38.963  **(0.010)** | 12.6 |
| Eastern Indo-Pacific | MHW days | 12.359  (**< 0.0001**) | 2001  (**0.0002**) | 1.308  (0.673) | 29.960  **(0.028)** | 22.9 |
|  | Events | 0.821  (**< 0.0001**) | 2001  (**0.0004**) | 0.276  (0.626) | 0.681  (0.239) | 2.5 |
|  | Mean intensity | -0.018  (**0.064**) | 2002  (0.176) | 0.007  (0.695) | -0.023  (0.347) | 3.3 |
|  | Max. intensity | -0.001  (0.991) | 1995  (0.772) | -0.210  **(0.037)** | -0.034  (0.359) | 0.2 |
|  | Cum. intensity | 15.143  (**< 0.0001**) | 2001  (**0.0005**) | 3.526  (0.315) | 30.542  (0.075) | 8.7 |
| Central Indo-Pacific | MHW days | 12.460  (**< 0.0001**) | 1997  (**< 0.0001**) | -1.560  (0.753) | 18.584  **(0.004)** | 11.9 |
|  | Events | 1.040  (**< 0.0001**) | 1997  (**< 0.0001**) | -0.260  (0.685) | 1.193  **(0.010)** | 4.6 |
|  | Mean intensity | -0.024  (**< 0.0001**) | 2002  (**0.0006**) | -0.009  (0.566) | -0.022  (0.112) | 2.3 |
|  | Max. intensity | 0.0005  (0.991) | 1984  (1) | -0.276  (1) | 0.008  (0.451) | 0.03 |
|  | Cum. intensity | 15.977  (**< 0.0001**) | 1997  (**< 0.0001**) | -2.185  (0.620) | 22.667  **(0.007)** | 10.4 |
| Western Indo-Pacific | MHW days | 13.529  (**< 0.0001**) | 2004  **(0.0001**) | 3.358  (0.369) | 35.480  **(0.010)** | 10.6 |
|  | Events | 1.097  (**< 0.0001**) | 2004  (**0.0006**) | 0.426  (0.342) | 1.808  **(0.041)** | 4.2 |
|  | Mean intensity | -0.015  **(0.017)** | 2006  **(0.021)** | 0.002  (0.907) | -0.002  (1) | 0.9 |
|  | Max. intensity | 0.011  (0.522) | 1995  (0.953) | -0.096  (0.125) | 0.006  (0.901) | 0.1 |
|  | Cum. intensity | 16.581  (**< 0.0001**) | 2004  (**0.0002**) | 4.369  (0.398) | 44.079  **(0.012)** | 10.1 |
| Tropical Eastern Pacific | MHW days | 6.569  **(0.008)** | 2005  (0.069) | 1.232  (0.785) | 23.978  (0.128) | 19.5 |
|  | Events | 0.620  **(0.008)** | 2001  (0.052) | -0.122  (0.820) | 1.271  (0.124) | 10.5 |
|  | Mean intensity | -0.018  (0.408) | 2016  (0.702) | 0.011  (0.532) | -0.366  **(0.009)** | 33 |
|  | Max. intensity | -0.007  (0.753) | 2017  (1) | 0.026  (0.438) | -0.521  (0.221) | 20 |
|  | Cum. intensity | 9.040  **(0.016)** | 2005  (0.103) | 1.333  (0.862) | 29.265  (0.174) | 22 |
| Temperate South America | MHW days | 5.471  **(0.013)** | 2003  (0.142) | 0.256  (0.910) | 16.635  (0.093) | 65 |
|  | Events | 0.240  (0.091) | 2003  (0.406) | -0.012  (0.910) | 0.420  (0.363) | 36.4 |
|  | Mean intensity | 0.047  **(0.018)** | 2006  **(0.015)** | 0.009  (0.761) | -0.083  (0.260) | 9.2 |
|  | Max. intensity | 0.091  **(0.003)** | 2006  **(0.002)** | 0.017  (0.761) | -0.075  (0.444) | 4.4 |
|  | Cum. intensity | 10.733  **(0.011)** | 2003  (0.125) | -0.716  (0.910) | 26.143  **(0.036)** | 36.5 |
| Temperate Southern Africa | MHW days | 8.778  (**< 0.0001**) | 1998  (**0.0003**) | 0.353  (0.837) | 11.928  **(0.004)** | 33.8 |
|  | Events | 0.667  (**< 0.0001**) | 1998  (**0.0002**) | -0.075  (0.773) | 0.650  **(0.031)** | 8.7 |
|  | Mean intensity | 0.052  (**0.001**) | 2005  **(0.013)** | 0.047  (0.385) | 0.050  (0.266) | 1.1 |
|  | Max. intensity | 0.113  (**< 0.0001**) | 2006  (**0.0004**) | 0.061  (0.338) | 0.093  (0.115) | 1.5 |
|  | Cum. intensity | 16.460  (**< 0.0001**) | 1998  (**0.0002**) | 3.146  (0.592) | 22.760  **(0.003)** | 7.2 |
| Temperate Australasia | MHW days | 13.940  (**< 0.0001**) | 1997  (**< 0.0001**) | -2.926  (0.499) | 16.179  (0.059) | 5.5 |
|  | Events | 0.914  (**< 0.0001**) | 1997  (**< 0.0001**) | -0.272  (0.558) | 0.692  (0.072) | 2.5 |
|  | Mean intensity | 0.012  (0.258) | 2008  **(0.032)** | -0.043  **(0.024)** | 0.004  (0.913) | 0.1 |
|  | Max. intensity | 0.063  **(0.011)** | 2007  (**0.0008**) | -0.055  (0.123) | 0.024  (0.692) | 0.4 |
|  | Cum. intensity | 20.956  (**< 0.0001**) | 1997  (**< 0.0001**) | -5.465  (0.444) | 25.350  **(0.042)** | 4.6 |
| Southern Ocean | MHW days | -4.672  (**0.0001**) | 2006  (**0.0003**) | -0.956  (0.414) | 0.409  (1) | 0.4 |
|  | Events | -0.482  (**< 0.0001**) | 2006  (**< 0.0001**) | -0.113  (0.338) | -0.291  (0.500) | 2.6 |
|  | Mean intensity | 0.104  (**< 0.0001**) | 2005  (**< 0.0001**) | -0.043  (0.056) | 0.253  **(0.002)** | 5.9 |
|  | Max. intensity | 0.139  (**< 0.0001**) | 2005  (**< 0.0001**) | -0.066  **(0.040)** | 0.341  **(0.001)** | 5.2 |
|  | Cum. intensity | -0.591  (0.492) | 1993  (0.490) | 7.170  (0.244) | 0.613  (0.722) | 0.1 |

**Table S4.** Trend of MHW Metrics in Global Coastal Realms per Season (Sen’s Slope*10 and Mann-Kendall p-value, and change-point detection).

| Realm | Season | Metric | Decadal Trend  (p-value) | Year Change Point  (p-value) | Trend Pre-Change Point  (p-value) | Trend Post-Change Point  (p-value) | Ratio Trends Post/Pre-Change Point |
| --- | --- | --- | --- | --- | --- | --- | --- |
| Arctic | Summer | MHW days | 4.004  (**< 0.0001**) | 2002  (**< 0.0001**) | 1.382  (0.156) | 6.307  **(0.025)** | 4.6 |
|  |  | Events | 0.191  (**< 0.0001**) | 2002  (**< 0.0001**) | 0.103  (0.239) | 0.057  (0.820) | 0.6 |
|  |  | Mean intensity | 0.359  (**< 0.0001**) | 2006  (**< 0.0001**) | 0.001  (0.981) | 0.268  (0.344) | 268.0 |
|  |  | Max. intensity | 0.555  (**< 0.0001**) | 2006  (**< 0.0001**) | -0.004  (0.907) | 0.463  (0.444) | 115.8 |
|  |  | Cum. intensity | 11.975  (**< 0.0001**) | 2002  (**< 0.0001**) | 2.326  (0.194) | 28.077  **(0.015)** | 12.1 |
|  | Autumn | MHW days | 4.895  (**< 0.0001**) | 2000  (**< 0.0001**) | 0.201  (0.78) | 9.28  **(0.001)** | 46.2 |
|  |  | Events | 0.248  (**< 0.0001**) | 2000  (1) | 0.067  (0.294) | 0.278  **(0.010)** | 4.1 |
|  |  | Mean intensity | 0.191  (**0.0001**) | 2002  (**0.0003**) | 0.087  (0.566) | 0.106  (0.538) | 1.2 |
|  |  | Max. intensity | 0.351  (**< 0.0001**) | 2002  (**< 0.0001**) | 0.066  (0.381) | 0.414  (0.127) | 6.3 |
|  |  | Cum. intensity | 8.001  (**< 0.0001**) | 2002  (**< 0.0001**) | 1.762  (0.065) | 16.354  **(0.015)** | 9.3 |
|  | Winter | MHW days | 3.778  (**< 0.0001**) | 2004  (**0.0001**) | -0.473  (0.635) | 13.157  **(0.049)** | 27.8 |
|  |  | Events | 0.179  (**0.0008**) | 2000  (**0.0002**) | -0.188  (0.08) | 0.166  (0.215) | -0.9 |
|  |  | Mean intensity | 0.086  (**< 0.0001**) | 1998  (**0.0004**) | 0.115  **(0.004)** | 0.02  (0.535) | 0.2 |
|  |  | Max. intensity | 0.144  (**< 0.0001**) | 1998  (**< 0.0001**) | 0.14  **(0.019)** | 0.04  (0.333) | 0.3 |
|  |  | Cum. intensity | 3.835  (**< 0.0001**) | 2000  (**< 0.0001**) | -0.174  (0.675) | 6.062  (**< 0.0001**) | 34.8 |
|  | Spring | Cumulative Intensity | 2.567  **(0.006)** | 2002  (**0.003**) | -1.257  **(0.037)** | 6.75  (0.086) | 5.4 |
|  |  | Maximum Intensity | 0.060  (0.118) | 2002  (**0.015**) | -0.087  (0.139) | -0.139  (0.284) | 1.6 |
|  |  | Duration | 0.055  **(0.032)** | 1989  (1) | 0.115  (0.536) | 0.003  (0.988) | 0.0 |
|  |  | Number of Events | 0.094  **(0.007)** | 1989  (**0.023**) | 0.158  (0.711) | 0.025  (0.566) | 0.2 |
|  |  |  | 2.458  (**< 0.0001**) | 2002  (**< 0.0001**) | 0.301  (0.216) | 3.702  (0.056) | 12.3 |
| Temperate Northern Atlantic | Summer | Cumulative Intensity | 4.860  (**< 0.0001**) | 2001  (**< 0.0001**) | 1.78  (0.086) | 4.846  **(0.028)** | 2.7 |
|  |  | Maximum Intensity | 0.348  (**< 0.0001**) | 2001  (**< 0.0001**) | 0.143  (0.064) | 0.319  **(0.043)** | 2.2 |
|  |  | Duration | 0.062  **(0.008)** | 2001  (**0.008**) | -0.038  (0.581) | 0.015  (0.651) | 0.4 |
|  |  | Number of Events | 0.196  (**< 0.0001**) | 2001  (**< 0.0001**) | -0.03  (0.77) | 0.143  (0.194) | 4.8 |
|  |  |  | 10.652  (**< 0.0001**) | 2001  (**< 0.0001**) | 4.312  (0.086) | 11.282  **(0.037)** | 2.6 |
|  | Autumn | MHW days | 5.735  (**< 0.0001**) | 1998  (**< 0.0001**) | 1.746  (0.108) | 9.288  **(0.001)** | 5.3 |
|  |  | Events | 0.342  (**< 0.0001**) | 1998  (**< 0.0001**) | 0.082  (0.266) | 0.371  **(0.002)** | 4.5 |
|  |  | Mean intensity | 0.008  (0.552) | 2011  (0.406) | -0.021  (0.254) | -0.021  (0.756) | 1.0 |
|  |  | Max. intensity | 0.071  **(0.005)** | 2009  (**0.005**) | -0.012  (0.737) | 0.134  (0.428) | 11.2 |
|  |  | Cum. intensity | 9.780  (**< 0.0001**) | 1998  (**< 0.0001**) | 2.411  (0.149) | 17.273  **(0.001)** | 7.2 |
|  | Winter | MHW days | 4.578  (**< 0.0001**) | 2001  (1) | 1.847  **(0.018)** | 6.814  **(0.002)** | 3.7 |
|  |  | Events | 0.342  (**< 0.0001**) | 2001  (**< 0.0001**) | 0.151  **(0.018)** | 0.342  **(0.010)** | 2.3 |
|  |  | Mean intensity | -0.012  (0.650) | 1993  (0.270) | 0.114  (0.732) | 0.043  (0.171) | 0.4 |
|  |  | Max. intensity | 0.045  (0.204) | 2009  (0.085) | -0.067  (0.072) | 0.204  (0.127) | 3.0 |
|  |  | Cum. intensity | 7.065  (**< 0.0001**) | 2004  (**< 0.0001**) | 3.09  **(0.002)** | 13.22  **(0.015)** | 4.3 |
|  | Spring | MHW days | 3.858  (**< 0.0001**) | 2001  (**< 0.0001**) | 1.971  (0.098) | 5.296  **(0.007)** | 2.7 |
|  |  | Events | 0.290  (**< 0.0001**) | 1997  (**< 0.0001**) | 0.064  (0.499) | 0.248  **(0.002)** | 3.9 |
|  |  | Mean intensity | -0.025  (0.316) | 1994  (0.161) | 0.027  (0.951) | 0.04  (0.213) | 1.5 |
|  |  | Max. intensity | 0.032  (0.376) | 2006  (0.097) | -0.193  **(0.023)** | 0.037  (0.964) | 0.2 |
|  |  | Cum. intensity | 6.493  (**< 0.0001**) | 1998  (**< 0.0001**) | 1.265  (0.303) | 9.134  **(0.003)** | 7.2 |
| Temperate Northern Pacific | Summer | MHW days | 4.837  (**< 0.0001**) | 2003  (**0.0006**) | 2.088  **(0.048)** | 9.218  (0.093) | 4.4 |
|  |  | Events | 0.352  (**< 0.0001**) | 2003  (**0.0007**) | 0.22  (0.063) | 0.412  (0.080) | 1.9 |
|  |  | Mean intensity | 0.067  **(0.002)** | 2005  (**0.0009**) | -0.017  (0.637) | 0.039  (0.592) | 2.3 |
|  |  | Max. intensity | 0.205  (**< 0.0001**) | 2003  (**< 0.0001**) | -0.068  (0.573) | 0.279  **(0.036)** | 4.1 |
|  |  | Cum. intensity | 11.247  (**< 0.0001**) | 2003  (**0.0004**) | 4.318  (0.055) | 23.789  **(0.050)** | 5.5 |
|  | Autumn | MHW days | 4.223  (**< 0.0001**) | 2002  (**0.0002**) | 1.161  (0.319) | 10.435  **(0.004)** | 9.0 |
|  |  | Events | 0.256  (**< 0.0001**) | 2002  (**0.0002**) | 0.109  (0.239) | 0.394  **(0.003)** | 3.6 |
|  |  | Mean intensity | 0.042  **(0.017)** | 2005  (**0.015**) | -0.016  (0.785) | -0.001  (1) | 0.1 |
|  |  | Max. intensity | 0.127  **(0.001)** | 2005  (**0.002**) | -0.004  (0.941) | 0.143  (0.387) | 35.8 |
|  |  | Cum. intensity | 7.627  (**< 0.0001**) | 2002  (**< 0.0001**) | 2.13  (0.216) | 20.921  **(0.003)** | 9.8 |
|  | Winter | MHW days | 2.210  **(0.0003)** | 1994  (**0.008**) | -0.801  (0.502) | 2.599  (0.051) | 3.2 |
|  |  | Events | 0.161  (**0.0001**) | 1994  (**< 0.004**) | -0.06  (0.502) | 0.166  **(0.046)** | 2.8 |
|  |  | Mean intensity | 0.049  **(0.005)** | 1997  (**< 0.005**) | 0.06  (0.26) | -0.016  (0.528) | 0.3 |
|  |  | Max. intensity | 0.099  (**< 0.0001**) | 1996  (**0.002**) | 0.115  (0.138) | 0.053  (0.290) | 0.5 |
|  |  | Cum. intensity | 3.386  (**< 0.0001**) | 1994  (**0.002**) | -0.763  (0.583) | 3.932  **(0.031)** | 5.2 |
|  | Spring | MHW days | 2.083  **(0.002)** | 2013  (**0.025**) | 0.418  (0.292) | -11.484  (0.755) | 27.5 |
|  |  | Events | 0.148  **(0.001)** | 2013  (**0.027**) | 0.05  (0.263) | 0.071  (0.917) | 1.4 |
|  |  | Mean intensity | 0.075  **(0.003)** | 1997  (**0.001**) | 0.015  (0.893) | -0.02  (0.624) | 1.3 |
|  |  | Max. intensity | 0.161  (**0.0001**) | 1997  (**0.0005**) | 0.073  (0.499) | 0.055  (0.498) | 0.8 |
|  |  | Cum. intensity | 3.881  (**0.0006**) | 1994  (**0.016**) | 1.521  (0.76) | 4.652  (0.119) | 3.1 |
| Tropical Atlantic | Summer | MHW days | 2.257  (**< 0.0001**) | 2002  (**0.0006**) | 0.575  (0.415) | 5.598  (0.112) | 9.7 |
|  |  | Events | 0.206  (**0.0002**) | 1997  (**0.003**) | -0.01  (0.964) | 0.26  (0.154) | 26.0 |
|  |  | Mean intensity | -0.031  **(0.010)** | 1996  (1) | -0.029  (0.692) | -0.017  (0.428) | 0.6 |
|  |  | Max. intensity | -0.007  (0.701) | 1984  (0.876) | 0.427  (0.296) | 0.007  (0.802) | 0.0 |
|  |  | Cum. intensity | 2.721  (**< 0.0001**) | 2002  (**0.0007**) | 0.653  (0.349) | 7.228  (0.074) | 11.1 |
|  | Autumn | MHW days | 4.610  (**< 0.0001**) | 2001  (**< 0.0001**) | 0.629  (0.256) | 9.994  **(0.010)** | 15.9 |
|  |  | Events | 0.384  (**< 0.0001**) | 2001  (**< 0.0001**) | 0.077  (0.206) | 0.398  **(0.032)** | 5.2 |
|  |  | Mean intensity | -0.023  **(0.033)** | 2002  (**0.034**) | 0.022  (0.45) | -0.013  (0.581) | 0.6 |
|  |  | Max. intensity | 0.003  (0.807) | 1994  (1) | -0.123  (0.36) | -0.011  (0.514) | 0.1 |
|  |  | Cum. intensity | 5.188  (**< 0.0001**) | 2001  (**< 0.0001**) | 0.652  (0.381) | 10.261  **(0.009)** | 15.7 |
|  | Winter | MHW days | 4.440  (**< 0.0001**) | 2001  (**< 0.0001**) | 0.448  (0.538) | 13.28  (**< 0.0001**) | 29.6 |
|  |  | Events | 0.365  (**< 0.0001**) | 2001  (**< 0.0001**) | 0.039  (0.538) | 0.711  **(0.002)** | 18.2 |
|  |  | Mean intensity | -0.030  **(0.014)** | 2002  **(0.012**) | 0.025  (0.415) | -0.017  (0.581) | 0.7 |
|  |  | Max. intensity | -0.007  (0.616) | 2002  (0.787) | 0.027  (0.608) | 0.054  (0.417) | 2.0 |
|  |  | Cum. intensity | 4.653  (**< 0.0001**) | 2001  (**< 0.0001**) | 0.656  (0.284) | 13.612  **(0.002)** | 20.8 |
|  | Spring | MHW days | 3.155  (**< 0.0001**) | 2001  (**0.0007**) | 0.236  (0.82) | 7.334  **(0.032)** | 31.1 |
|  |  | Events | 0.229  (**< 0.0001**) | 2001  (**< 0.0005**) | 0.044  (0.673) | 0.395  (0.075) | 9.0 |
|  |  | Mean intensity | -0.039  **(0.003)** | 2002  (**0.014**) | -0.011  (0.74) | -0.03  (0.315) | 2.7 |
|  |  | Max. intensity | -0.033  (0.188) | 1998  (0.378) | -0.01  (0.902) | 0.018  (0.862) | 1.8 |
|  |  | Cum. intensity | 3.660  (**< 0.0001**) | 2001  (**0.001**) | 0.112  (0.974) | 8.258  **(0.037)** | 73.7 |
| Eastern Indo-Pacific | Summer | MHW days | 3.094  (**0.0001**) | 2001  (**0.002**) | 0.097  (0.922) | 6.408  **(0.037)** | 66.1 |
|  |  | Events | 0.200  (**0.0007**) | 2001  **(0.002)** | 0.017  (0.871) | 0.186  (0.415) | 10.9 |
|  |  | Mean intensity | -0.011  (0.408) | 2012  (0.967) | 0.008  (0.734) | -0.033  (0.592) | 4.1 |
|  |  | Max. intensity | 0.026  (0.159) | 1995  (0.171) | -0.156  (0.101) | 0.006  (0.835) | 0.0 |
|  |  | Cum. intensity | 3.897  (**< 0.0002**) | 2001  **(0.004)** | 0.152  (0.974) | 7.145  **(0.032)** | 47.0 |
|  | Autumn | MHW days | 2.833  (**< 0.0001**) | 2001  **(0.002)** | 0.032  (0.871) | 10.561  **(0.020)** | 330.0 |
|  |  | Events | 0.202  **(0.001)** | 2001  **(0.005)** | -0.029  (0.82) | 0.291  (0.174) | 10.0 |
|  |  | Mean intensity | -0.023  **(0.041)** | 2002  (0.235) | -0.013  (0.88) | -0.034  (0.284) | 2.6 |
|  |  | Max. intensity | -0.011  (0.584) | 1999  (1) | 0.019  (0.762) | 0.003  (0.958) | 0.2 |
|  |  | Cum. intensity | 3.366  (**0.0002**) | 2001  **(0.003)** | 0.094  (0.871) | 11.414  **(0.050)** | 121.4 |
|  | Winter | MHW days | 3.511  (**< 0.0001**) | 1998  (**0.0005**) | 1.9  (0.149) | 6.61  **(0.009)** | 3.5 |
|  |  | Events | 0.241  (**< 0.0001**) | 2001  (**0.0009**) | 0.196  **(0.041)** | 0.243  (0.110) | 1.2 |
|  |  | Mean intensity | -0.015  (0.239) | 2002  (0.222) | 0.054  (0.11) | -0.01  (0.538) | 0.2 |
|  |  | Max. intensity | 0.005  (0.801) | 2002  (1) | 0.106  (0.065) | 0.031  (0.496) | 0.3 |
|  |  | Cum. intensity | 3.648  (**< 0.0001**) | 1998  (**0.0007**) | 2.042  (0.174) | 6.693  **(0.012)** | 3.3 |
|  | Spring | MHW days | 2.949  (**< 0.0001**) | 2001  (**0.0003**) | 0.35  (0.626) | 4.479  **(0.037)** | 12.8 |
|  |  | Events | 0.176  (**0.0002**) | 2000  (**0.0005**) | -0.005  (1) | 0.102  (0.284) | 20.4 |
|  |  | Mean intensity | -0.018  (0.130) | 2002  (0.176) | 0.029  (0.45) | -0.016  (0.721) | 0.6 |
|  |  | Max. intensity | -0.012  (0.753) | 1995  (0.831) | -0.297  (0.016) | -0.06  (0.211) | 0.2 |
|  |  | Cum. intensity | 3.657  (**< 0.0001**) | 1999  (0.001) | 0.027  (1) | 5.316  **(0.013)** | 196.9 |
| Central Indo-Pacific | Summer | MHW days | 3.386  (**< 0.0001**) | 2000  (**0.0002**) | 0.577  (0.675) | 6.747  **(0.011)** | 11.7 |
|  |  | Events | 0.279  (**< 0.0001**) | 1997  (**0.0004**) | -0.057  (0.62) | 0.327  **(0.024)** | 5.7 |
|  |  | Mean intensity | -0.027  (**0.0005**) | 2006  (**0.004**) | -0.009  (0.528) | -0.023  (0.344) | 2.6 |
|  |  | Max. intensity | 0.004  (0.935) | 1994  (0.938) | -0.198  **(0.017)** | -0.012  (0.707) | 0.1 |
|  |  | Cum. intensity | 4.535  (**< 0.0001**) | 1997  (**0.0003**) | -0.734  (0.62) | 7.054  **(0.007)** | 9.6 |
|  | Autumn | MHW days | 3.055  (**< 0.0001**) | 1997  (**0.0001**) | -0.322  (0.62) | 4.437  **(0.014)** | 13.8 |
|  |  | Events | 0.260  (**< 0.0001**) | 1997  (**0.0003**) | -0.044  (0.685) | 0.274  (0.065) | 6.2 |
|  |  | Mean intensity | -0.021  **(0.003)** | 2006  (**0.007**) | -0.004  (0.726) | -0.002  (0.964) | 0.5 |
|  |  | Max. intensity | -0.004  (0.735) | 2006  (0.816) | 0.026  (0.272) | 0.038  (0.620) | 1.5 |
|  |  | Cum. intensity | 3.731  (**< 0.0001**) | 1997  (**0.0003**) | -0.6  (0.558) | 4.796  **(0.030)** | 8.0 |
|  | Winter | MHW days | 3.224  (**< 0.0001**) | 1997  (**< 0.0001**) | 0.28  (0.753) | 4.939  **(0.002)** | 17.6 |
|  |  | Events | 0.274  (**< 0.0001**) | 1997  (**< 0.0001**) | -0.003  (1) | 0.334  **(0.005)** | 111.3 |
|  |  | Mean intensity | -0.012  (0.079) | 2002  (**0.034**) | 0.002  (0.74) | 0.012  (0.315) | 6.0 |
|  |  | Max. intensity | 0.017  (0.067) | 2009  (0.134) | -0.011  (0.514) | 0.062  (0.127) | 5.6 |
|  |  | Cum. intensity | 4.080  (**< 0.0001**) | 1997  (**< 0.0001**) | 0.339  (0.822) | 5.587  **(0.002)** | 16.5 |
|  | Spring | MHW days | 3.010  (**< 0.0001**) | 1997  (**0.0003**) | -0.747  (0.392) | 4.625  **(0.008)** | 6.2 |
|  |  | Events | 0.264  (**< 0.0001**) | 1997  (**0.0003**) | -0.091  (0.392) | 0.345  **(0.047)** | 3.8 |
|  |  | Mean intensity | -0.026  **(0.004)** | 2000  (**0.031**) | -0.008  (0.78) | -0.022  (0.259) | 2.8 |
|  |  | Max. intensity | -0.003  (0.789) | 1983  (1) | -0.041  (1) | 0.004  (0.885) | 0.1 |
|  |  | Cum. intensity | 3.732  (**< 0.0001**) | 1997  (**0.0003**) | -1.119  (0.344) | 5.699  **(0.016)** | 5.1 |
| Western Indo-Pacific | Summer | MHW days | 3.197  (**< 0.0001**) | 2004  (**0.0004**) | 0.947  (0.291) | 10.495  **(0.010)** | 11.1 |
|  |  | Events | 0.309  (**< 0.0001**) | 2004  (**0.0005**) | 0.121  (0.316) | 0.543  **(0.028)** | 4.5 |
|  |  | Mean intensity | -0.008  (0.204) | 2007  (0.204) | 0.014  (0.311) | -0.007  **(0.767)** | 0.5 |
|  |  | Max. intensity | 0.037  (0.055) | 1996  (0.222) | -0.046  (0.692) | 0.027  (0.481) | 0.6 |
|  |  | Cum. intensity | 4.253  (**< 0.0001**) | 2004  (**0.0005**) | 1.306  (0.267) | 14.34  **(0.012)** | 11.0 |
|  | Autumn | MHW days | 3.138  (**< 0.0001**) | 2004  (**0.0005**) | 1.31  (0.154) | 7.429  (0.058) | 5.7 |
|  |  | Events | 0.269  (**< 0.0001**) | 1996  (1) | -0.218  (0.488) | 0.36  **(0.009)** | 1.7 |
|  |  | Mean intensity | -0.016  (0.127) | 2005  (0.216) | 0.009  (0.71) | -0.007  (0.902) | 0.8 |
|  |  | Max. intensity | 0.012  (0.568) | 1996  (1) | -0.117  (0.553) | -0.008  (0.826) | 0.1 |
|  |  | Cum. intensity | 3.739  (**< 0.0001**) | 2004  (**< 0.0008**) | 1.721  (0.154) | 9.125  (0.069) | 5.3 |
|  | Winter | MHW days | 2.956  (**< 0.0001**) | 2005  (**0.0003**) | 0.711  (0.264) | 6.194  **(0.044)** | 8.7 |
|  |  | Events | 0.235  (**< 0.0001**) | 2004  (**0.0008**) | 0.08  (0.342) | 0.364  (0.096) | 4.6 |
|  |  | Mean intensity | -0.011  (0.079) | 2001  (0.151) | 0.024  (0.23) | -0.021  (0.239) | 0.9 |
|  |  | Max. intensity | 0.009  (0.701) | 1985  (1) | 0.078  (1) | 0.02  (0.255) | 0.3 |
|  |  | Cum. intensity | 3.358  (**< 0.0001**) | 2005  (**0.0004**) | 0.885  (0.309) | 7.146  **(0.044)** | 8.1 |
|  | Spring | MHW days | 3.570  (**< 0.0001**) | 2004  (1) | 0.804  (0.369) | 11.28  **(0.008)** | 14.0 |
|  |  | Events | 0.284  (**< 0.0001**) | 2001  (**0.0005**) | 0.008  (0.974) | 0.434  **(0.005)** | 54.3 |
|  |  | Mean intensity | -0.024  **(0.009)** | 2001  (**0.005**) | 0.022  (0.456) | -0.008  (0.740) | 0.4 |
|  |  | Max. intensity | 0.000  (0.972) | 1985  (0.661) | -0.225  (0.734) | 0.014  (0.255) | 0.1 |
|  |  | Cum. intensity | 4.180  (**< 0.0001**) | 2004  (**0.0002**) | 0.942  (0.245) | 12.943  **(0.012)** | 13.7 |
| Tropical Eastern Pacific | Summer | MHW days | 1.593  **(0.009)** | 1996  (0.057) | -1.652  (0.488) | 2.193  (0.201) | 1.3 |
|  |  | Events | 0.155  **(0.013)** | 1996  (**0.050**) | -0.327  (0.373) | 0.181  (0.270) | 0.6 |
|  |  | Mean intensity | -0.010  (0.507) | 1984  (1) | -0.785  (1) | 0.002  (0.900) | 0.0 |
|  |  | Max. intensity | 0.001  (0.991) | 1984  (0.816) | -2.009  (1) | 0.034  (0.365) | 0.0 |
|  |  | Cum. intensity | 2.212  **(0.011)** | 1996  (0.007) | -2.188  (0.488) | 2.719  (0.201) | 1.2 |
|  | Autumn | MHW days | 1.732  **(0.007)** | 1996  (0.087) | -0.293  (0.621) | 3.708  (0.094) | 12.7 |
|  |  | Events | 0.170  **(0.008)** | 2011  (0.084) | 0.048  (0.372) | 0.192  (0.876) | 4.0 |
|  |  | Mean intensity | -0.046  **(0.044)** | 2011  (0.285) | -0.019  (0.721) | -0.173  (0.350) | 9.1 |
|  |  | Max. intensity | -0.058  (0.159) | 2002  (0.816) | -0.009  (0.928) | -0.093  (0.315) | 10.3 |
|  |  | Cum. intensity | 2.013  **(0.011)** | 1996  (0.100) | -0.488  (0.621) | 4.462  (0.146) | 9.1 |
|  | Winter | MHW days | 1.581  **(0.009)** | 2013  (0.055) | 0.355  (0.593) | -1.776  (1) | 5.0 |
|  |  | Events | 0.145  **(0.010)** | 2013  (0.060) | 0.049  (0.549) | -0.427  (0.755) | 8.7 |
|  |  | Mean intensity | 0.023  (0.449) | 2001  (0.426) | -0.104  (0.206) | -0.013  (0.928) | 0.1 |
|  |  | Max. intensity | 0.034  (0.311) | 2006  (0.378) | -0.033  (0.624) | -0.06  (0.558) | 1.8 |
|  |  | Cum. intensity | 2.312  **(0.016)** | 2013  (0.090) | 0.636  (0.593) | -8.539  (0.917) | 13.4 |
|  | Spring | MHW days | 0.999  (0.121) | 2001  (0.387) | -0.564  (0.77) | 2.897  (0.415) | 5.1 |
|  |  | Events | 0.092  (0.159) | 2001  (0.285) | -0.128  (0.456) | 0.163  (0.608) | 1.3 |
|  |  | Mean intensity | -0.008  (0.735) | 1995  (1) | -0.187  (0.324) | -0.083  (0.243) | 0.4 |
|  |  | Max. intensity | -0.001  (0.972) | 1983  (1) | 4.102  (1) | 0.015  (0.735) | 0.0 |
|  |  | Cum. intensity | 1.274  (0.213) | 2001  (0.513) | -0.986  (0.673) | 2.292  (0.566) | 2.3 |
| Temperate  South  America | Summer | MHW days | 1.385  **(0.041)** | 2004  (**0.040**) | -1.538  (0.398) | 1.232  (0.545) | 0.8 |
|  |  | Events | 0.059  (0.213) | 2004  (0.100) | -0.113  (0.154) | -0.032  (0.820) | 0.3 |
|  |  | Mean intensity | 0.050  **(0.017)** | 2007  (**0.017**) | 0.009  (0.791) | -0.046  (0.322) | 5.1 |
|  |  | Max. intensity | 0.105  **(0.003)** | 2007  (**0.003**) | 0.024  (0.791) | -0.095  (0.488) | 4.0 |
|  |  | Cum. intensity | 2.545  **(0.049)** | 2004  (**0.003**) | -2.849  (0.291) | 2.132  (0.649) | 0.7 |
|  | Autumn | MHW days | 2.042  **(0.001)** | 2003  (**0.050**) | 0.962  (0.367) | 4.289  (0.080) | 4.5 |
|  |  | Events | 0.118  **(0.015)** | 1996  (0.241) | 0.008  (1) | 0.136  (0.158) | 17.0 |
|  |  | Mean intensity | 0.061  **(0.032)** | 2006  (0.079) | 0.023  (0.691) | -0.04  (0.753) | 1.7 |
|  |  | Max. intensity | 0.126  **(0.004)** | 2006  (**0.016**) | 0.051  (0.559) | 0.034  (0.753) | 0.7 |
|  |  | Cum. intensity | 3.410  **(0.001)** | 2003  (**0.046**) | 1.242  (0.43) | 8.269  (0.059) | 6.7 |
|  | Winter | MHW days | 1.687  **(0.014)** | 2013  (0.093) | 0.227  (0.661) | 9.369  (0.466) | 41.3 |
|  |  | Events | 0.112  (0.053) | 2014  (0.270) | 0.015  (0.816) | 0.551  (0.387) | 36.7 |
|  |  | Mean intensity | 0.019  (0.456) | 2006  (0.350) | -0.026  (0.624) | -0.171  (0.096) | 6.6 |
|  |  | Max. intensity | 0.058  (0.155) | 2000  (0.125) | -0.122  (0.263) | 0.027  (1) | 0.2 |
|  |  | Cum. intensity | 2.668  **(0.017)** | 2013  (0.156) | 0.435  (0.593) | 5.372  (0.252) | 12.3 |
|  | Spring | MHW days | 0.844  (0.067) | 2013  (0.134) | -0.138  (0.833) | 4.643  (0.466) | 33.6 |
|  |  | Events | 0.020  (0.568) | 2013  (0.536) | -0.046  (0.307) | 0.138  (0.917) | 3.0 |
|  |  | Mean intensity | 0.039  **(0.035)** | 2006  **(0.021)** | -0.015  (0.691) | -0.047  (0.685) | 3.1 |
|  |  | Max. intensity | 0.083  **(0.026)** | 2006  (**0.018**) | -0.038  (0.559) | 0.014  (0.893) | 0.4 |
|  |  | Cum. intensity | 1.691  **(0.019)** | 2010  (0.006) | -0.124  (0.896) | 10.716  (0.115) | 86.4 |
| Temperate Southern Africa | Summer | MHW days | 3.218  (**< 0.0001**) | 1998  (**< 0.0001**) | 1.43  (0.108) | 2.774  (0.130) | 1.9 |
|  |  | Events | 0.275  (**< 0.0001**) | 1998  (**< 0.0001**) | 0.157  (0.053) | 0.22  (0.112) | 1.4 |
|  |  | Mean intensity | 0.026  (0.159) | 2007  (0.204) | -0.014  (0.597) | -0.015  (0.921) | 1.1 |
|  |  | Max. intensity | 0.092  **(0.002)** | 2006  (**0.011**) | 0.024  (0.624) | 0.095  (0.344) | 4.0 |
|  |  | Cum. intensity | 6.152  (**< 0.0001**) | 2001  (**< 0.0001**) | 2.926  (0.056) | 6.315  (0.264) | 2.2 |
|  | Autumn | MHW days | 1.976  **(0.001)** | 2009  (**0.040**) | 0.848  (0.277) | 7.271  (0.161) | 8.6 |
|  |  | Events | 0.145  **(0.0004)** | 1998  (**0.005**) | -0.054  (0.65) | 0.19  **(0.037)** | 3.5 |
|  |  | Mean intensity | 0.027  (0.176) | 2005  (0.457) | 0.017  (0.823) | -0.048  (0.651) | 2.8 |
|  |  | Max. intensity | 0.067  (0.098) | 2006  (0.204) | -0.004  (0.944) | -0.002  (1) | 0.5 |
|  |  | Cum. intensity | 3.413  **(0.002)** | 2009  (**0.050**) | 1.243  (0.333) | 13.863  (0.161) | 11.2 |
|  | Winter | MHW days | 1.972  **(0.002)** | 2004  (**0.012**) | 0.346  (0.792) | 4.756  (0.173) | 13.7 |
|  |  | Events | 0.122  **(0.004)** | 1997  (**0.015**) | -0.156  (0.163) | 0.163  (0.080) | 1.0 |
|  |  | Mean intensity | 0.063  **(0.001)** | 2002  (**0.026**) | 0.07  (0.194) | 0.048  (0.381) | 0.7 |
|  |  | Max. intensity | 0.129  **(0.0002)** | 2006  (**0.007**) | 0.085  (0.129) | 0.113  **(0.500)** | 1.3 |
|  |  | Cum. intensity | 3.152  **(0.001)** | 2004  (**0.011**) | 0.792  (0.561) | 7.475  (0.173) | 9.4 |
|  | Spring | MHW days | 1.442  **(0.005)** | 2002  (**0.012**) | -0.174  (0.74) | 2.037  (0.230) | 11.7 |
|  |  | Events | 0.104  **(0.020)** | 2002  (**0.019**) | -0.047  (0.566) | 0.067  (0.721) | 1.4 |
|  |  | Mean intensity | 0.047  **(0.017)** | 2003  (**0.074**) | 0.015  (0.91) | 0.036  (0.401) | 2.4 |
|  |  | Max. intensity | 0.099  **(0.001)** | 2003  (**0.011**) | 0.066  (0.463) | 0.087  (0.442) | 1.3 |
|  |  | Cum. intensity | 2.663  **(0.001)** | 2002  (**0.006**) | 0.201  (0.928) | 3.179  (0.256) | 15.8 |
| Temperate Australasia | Summer | MHW days | 3.410  (**< 0.0001**) | 1997  (**< 0.0001**) | -0.856  (0.499) | 4.49  **(0.002)** | 5.2 |
|  |  | Events | 0.250  (**< 0.0001**) | 2004  (**0.0001**) | 0.102  (0.205) | 0.414  **(0.008)** | 4.1 |
|  |  | Mean intensity | 0.024  (0.083) | 2009  (0.134) | -0.013  (0.621) | 0.052  (0.502) | 4.0 |
|  |  | Max. intensity | 0.087  **(0.006)** | 2008  (**0.006**) | -0.012  (0.835) | 0.079  (0.743) | 6.6 |
|  |  | Cum. intensity | 6.272  (**< 0.0001**) | 1997  (**0.0001**) | -2.033  (0.392) | 8.82  **(0.002)** | 4.3 |
|  | Autumn | MHW days | 3.926  (**< 0.0001**) | 1997  (**0.0001**) | -0.446  (0.753) | 4.485  (0.065) | 10.1 |
|  |  | Events | 0.229  (**< 0.0001**) | 1997  (**0.0002**) | -0.054  (0.558) | 0.161  (0.080) | 3.0 |
|  |  | Mean intensity | 0.003  (0.834) | 2007  (0.622) | -0.044  (0.064) | 0.005  (1) | 0.1 |
|  |  | Max. intensity | 0.050  (0.059) | 2006  (**0.017**) | -0.088  (0.183) | 0.033  (0.753) | 0.4 |
|  |  | Cum. intensity | 6.193  (**< 0.0001**) | 1997  (**0.0001**) | -0.845  (0.558) | 6.982  (0.097) | 8.3 |
|  | Winter | MHW days | 3.461  (**< 0.0001**) | 1997  (**< 0.0001**) | -0.329  (0.753) | 1.283  (0.388) | 3.9 |
|  |  | Events | 0.213  **(0.0002)** | 1997  (**0.0001**) | -0.031  (0.822) | 0.035  (0.726) | 1.1 |
|  |  | Mean intensity | 0.007  (0.470) | 2005  (0.342) | -0.02  (0.309) | -0.007  (0.484) | 0.4 |
|  |  | Max. intensity | 0.049  **(0.009)** | 2005  (**0.017**) | 0.005  (0.901) | 0.026  (0.902) | 5.2 |
|  |  | Cum. intensity | 4.647  (**< 0.0001**) | 1997  (**< 0.0001**) | -0.374  (0.822) | 2.425  (0.498) | 6.5 |
|  | Spring | MHW days | 2.581  (**0.0002**) | 1997  (**0.0008**) | -0.948  (0.344) | 2.638  (0.141) | 2.8 |
|  |  | Events | 0.185  **(0.001)** | 1997  (**0.002**) | -0.091  (0.3) | 0.108  (0.469) | 1.2 |
|  |  | Mean intensity | 0.014  (0.289) | 2006  (0.107) | -0.052  **(0.034)** | 0.053  (0.392) | 1.0 |
|  |  | Max. intensity | 0.064  **(0.031)** | 2006  (**0.011**) | -0.043  (0.252) | 0.066  (0.620) | 1.5 |
|  |  | Cum. intensity | 3.938  (**0.0006**) | 1997  (**0.001**) | -1.353  (0.224) | 4.077  (0.183) | 3.0 |
| Southern Ocean | Summer | MHW days | -0.924  **(0.016)** | 1997  (0.090) | 0.32  (0.964) | -1.072  (0.272) | 3.4 |
|  |  | Events | -0.094  (**0.0006**) | 1993  (**0.011**) | 0.14  (0.451) | -0.086  (0.075) | 0.6 |
|  |  | Mean intensity | 0.010  (0.435) | 2016  (0.121) | -0.015  (0.14) | 0.202  (0.707) | 13.5 |
|  |  | Max. intensity | 0.007  (0.718) | 2016  (0.147) | -0.045  (0.057) | 0.391  (0.707) | 8.7 |
|  |  | Cum. intensity | -0.888  (0.055) | 1993  (0.110) | 4.009  (0.244) | -0.547  (0.561) | 0.1 |
|  | Autumn | MHW days | -0.591  (0.059) | 1995  (0.147) | 0.357  (0.827) | -0.258  (0.803) | 0.7 |
|  |  | Events | -0.076  **(0.002)** | 1994  (**0.010**) | 0.035  (0.541) | -0.042  (0.295) | 1.2 |
|  |  | Mean intensity | 0.078  (**0.0004**) | 2005  (1) | -0.038  (0.107) | 0.211  **(0.006)** | 5.6 |
|  |  | Max. intensity | 0.104  (**0.0007**) | 2005  (**0.005**) | -0.074  (0.078) | 0.296  **(0.002)** | 4.0 |
|  |  | Cum. intensity | 0.187  (0.470) | 2008  (0.151) | -0.591  **(0.033)** | 0.659  (0.584) | 1.1 |
|  | Winter | MHW days | -1.591  (**0.0003**) | 2006  (**0.0002**) | -0.299  (0.591) | 1.078  (0.392) | 3.6 |
|  |  | Events | -0.140  (**< 0.0001**) | 2005  (**< 0.0001**) | -0.018  (0.673) | -0.03  (0.651) | 1.7 |
|  |  | Mean intensity | 0.111  (**0.0001**) | 2005  (**< 0.0001**) | -0.028  (0.087) | 0.218  (0.053) | 7.8 |
|  |  | Max. intensity | 0.157  (**0.0001**) | 2005  (**< 0.0001**) | -0.04  (0.078) | 0.302  (0.064) | 7.6 |
|  |  | Cum. intensity | 0.197  (0.155) | 2008  (0.055) | -0.285  (0.095) | 0.736  (0.324) | 2.6 |
|  | Spring | MHW days | -1.527  (**0.0005**) | 2005  (**< 0.0001**) | 0.774  (0.107) | -0.964  (0.434) | 1.2 |
|  |  | Events | -0.144  (**0.0002**) | 2005  (**< 0.0001**) | 0.053  (0.118) | -0.087  (0.303) | 1.6 |
|  |  | Mean intensity | 0.143  (**< 0.0001**) | 2005  (**< 0.0001**) | -0.024  (0.413) | 0.362  **(0.001)** | 15.1 |
|  |  | Max. intensity | 0.197  (**< 0.0001**) | 2005  (**< 0.0001**) | -0.036  (0.442) | 0.499  **(0.001)** | 13.9 |
|  |  | Cum. intensity | -0.074  (0.666) | 2002  (0.675) | 0.485  (0.156) | 0.198  (0.673) | 0.4 |

**Figure S7.** Map showing the direction of trend from the seasonal trend analyses on MHW days, number of events, mean intensity, maximum intensity, and cumulative intensity (bars from left to right; 4 seasonal analyses per metric) at a global scale (insert) and for 12 coastal realms (map) ^1^. Of the 240 analyses (12 regions x 5 metrics x 4 seasons, see Fig. 3-6), 60% increased significantly, 9% decreased and 31% were unaffected. Only the most coastal OISST pixels within the realms were kept in the analysis (n=16,160). Map and figures were generated using the R free software environment (version 4.1.0, <https://www.r-project.org/>).


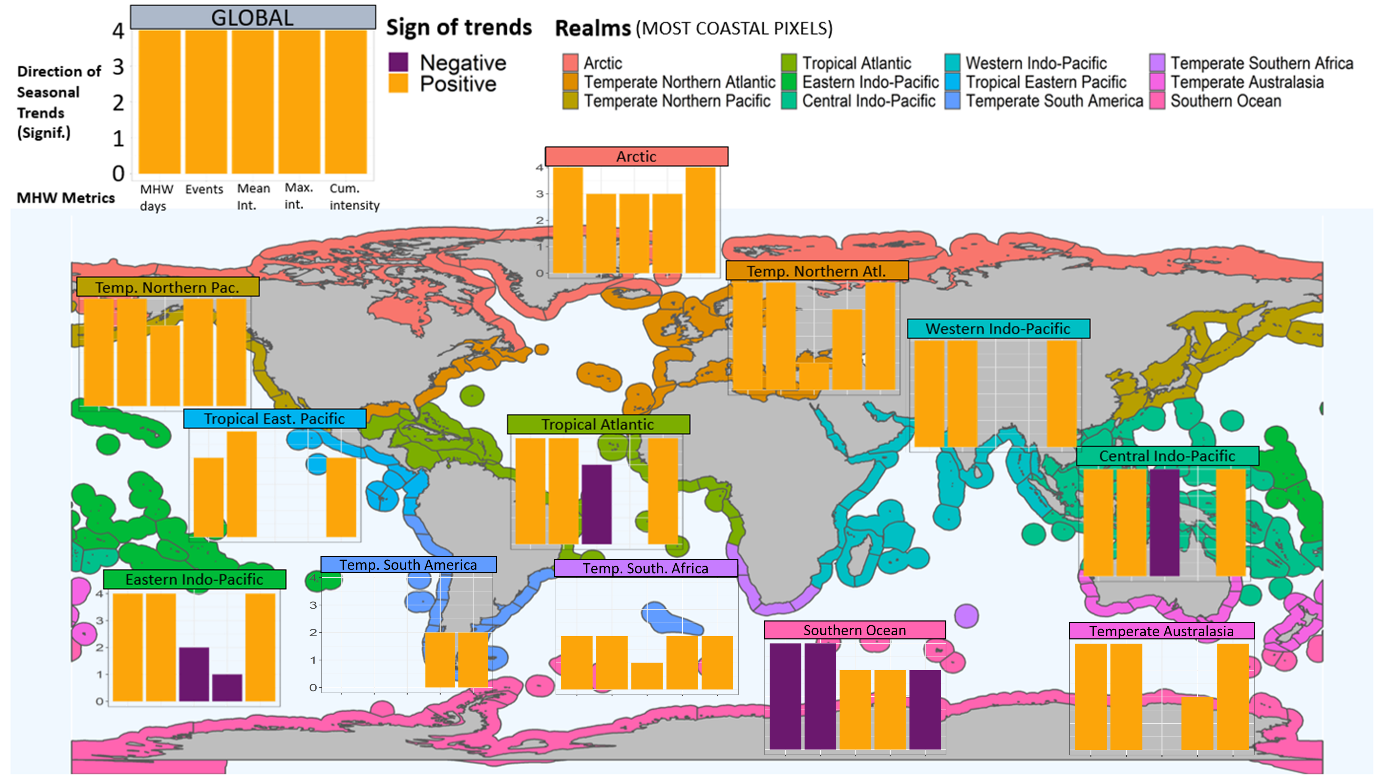


**Figure S8.** Global trends from January 1982 to December 2021 in the number of yearly-averaged and pixel-averaged marine heatwave days, number of events, mean intensity (°C) , maximum intensity (°C) and cumulative intensity (°C days) of marine heatwaves in the most coastal OISST pixels within coastal realms. See caption of figure S1 for similarities in figure legend.


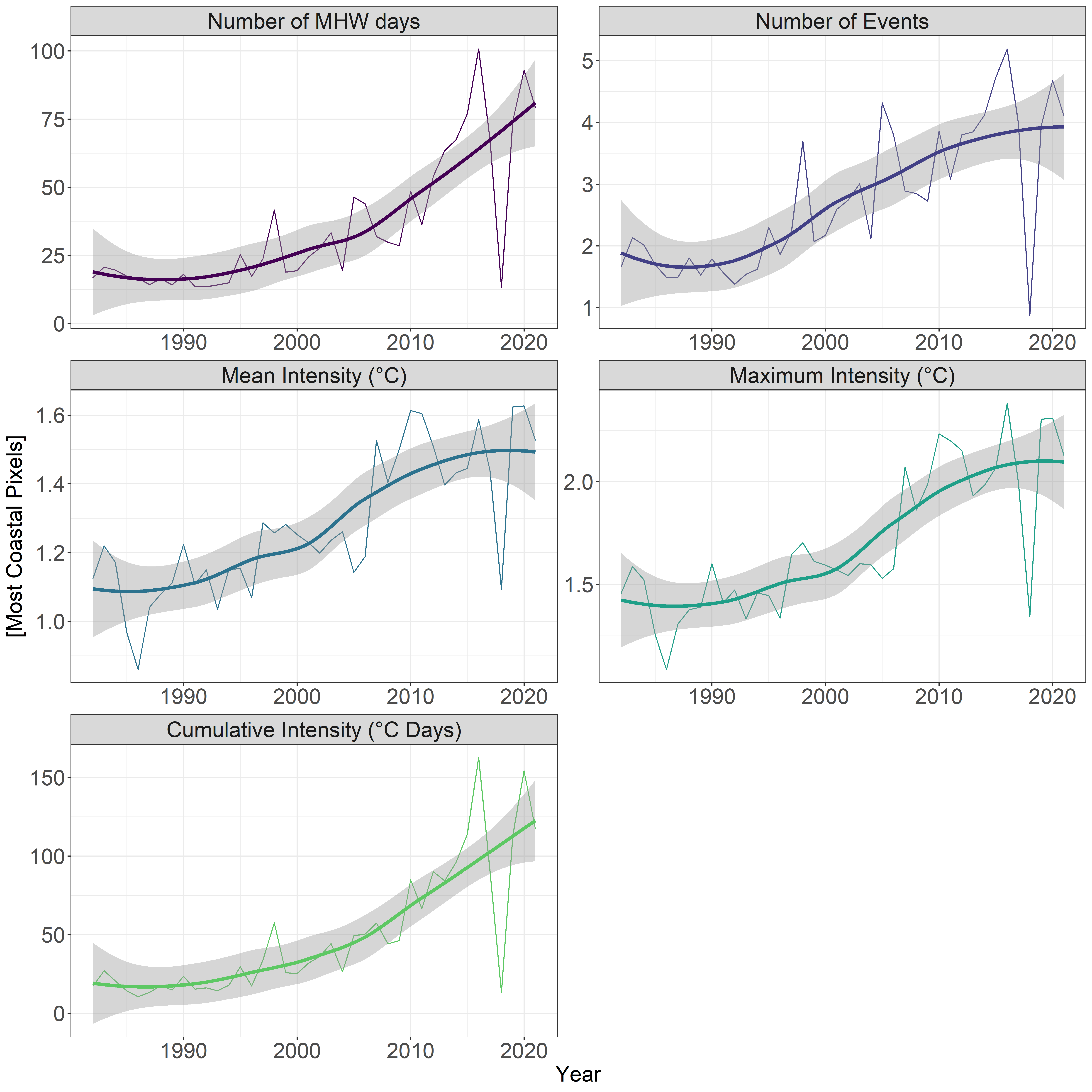


**Figure S9.** Seasonal trends from 1982 to 2021 in the yearly-averaged and pixel-averaged marine heatwave days per coastal realm and season. Only the most coastal OISST pixels have been used here. See caption of figure 2 for similarities in figure legend.


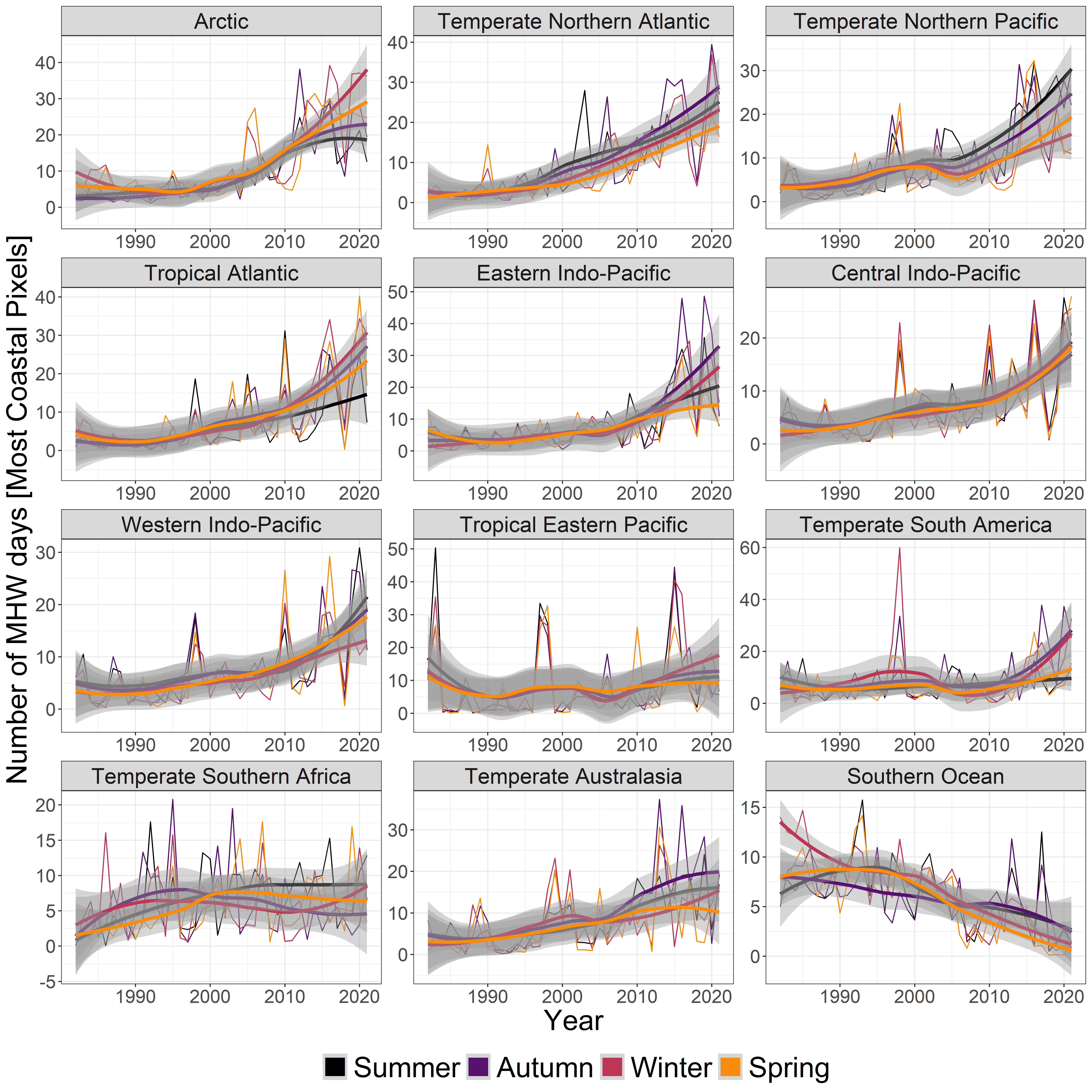


**Figure S10.** Seasonal trends from 1982 to 2021 in the yearly-averaged and pixel-averaged number of marine heatwaves (events) per coastal realm and season. Only the most coastal OISST pixels have been used here. See caption of figure 2 for similarities in figure legend.


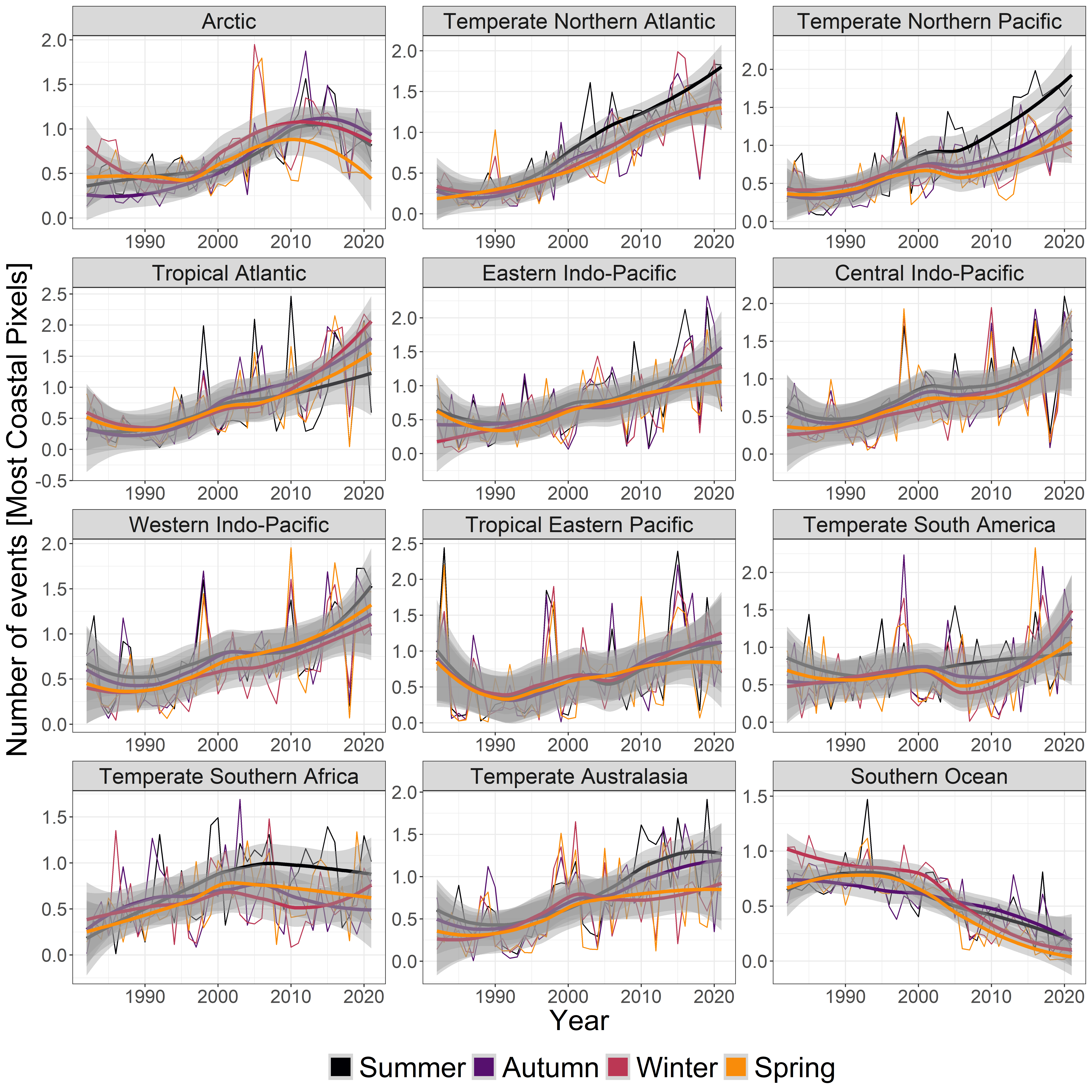


**Figure S11.** Seasonal trends from 1982 to 2021 in the yearly-averaged and pixel-averaged mean intensity (°C) of marine heatwaves per coastal realm and season. Only the most coastal OISST pixels have been used here. See caption of figure 2 for similarities in figure legend.


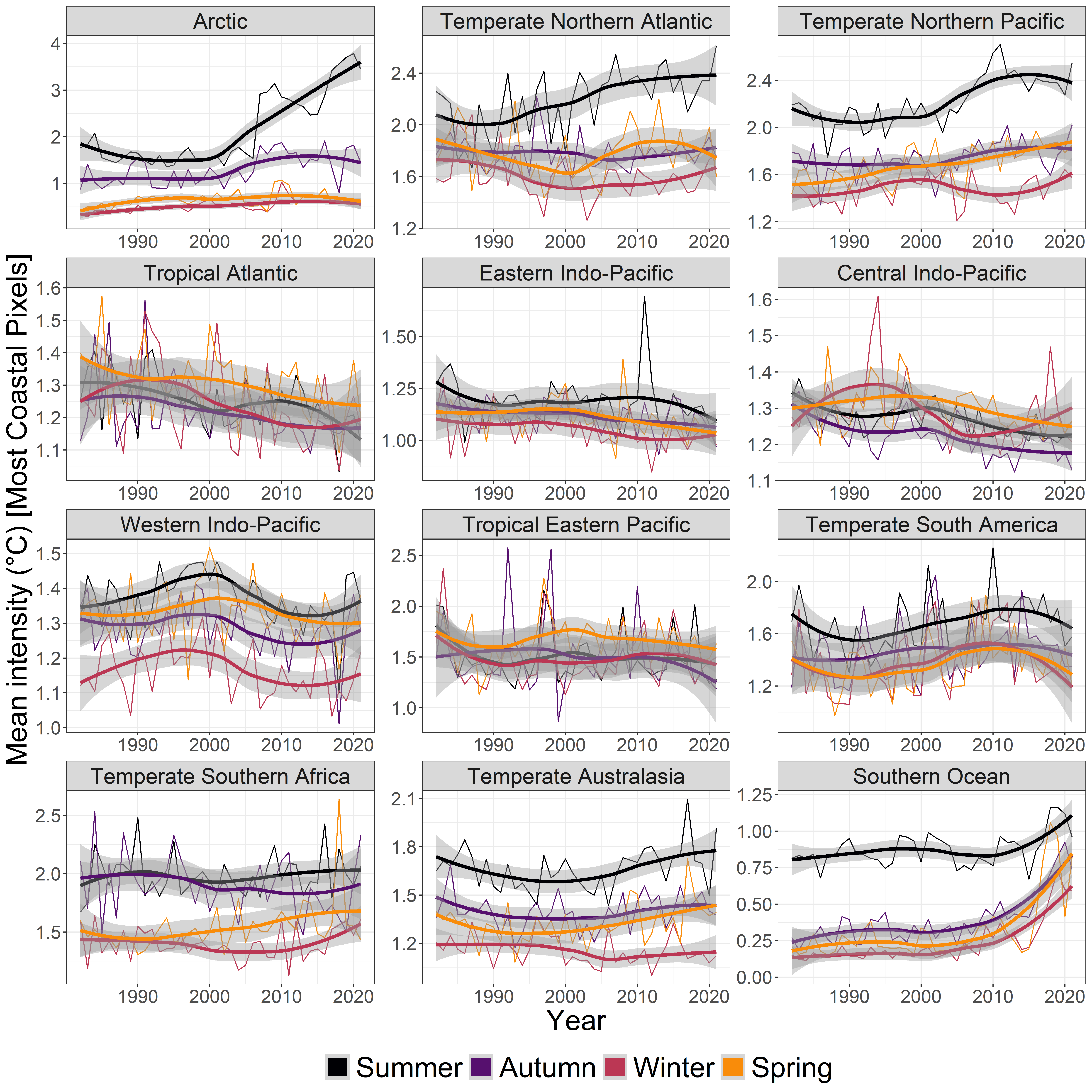


**Figure S12.** Seasonal trends from 1982 to 2021 in the yearly-averaged and pixel-averaged maximum intensity (°C) of marine heatwaves per coastal realm and season. Only the most coastal OISST pixels have been used here. See caption of figure 2 for similarities in figure legend.


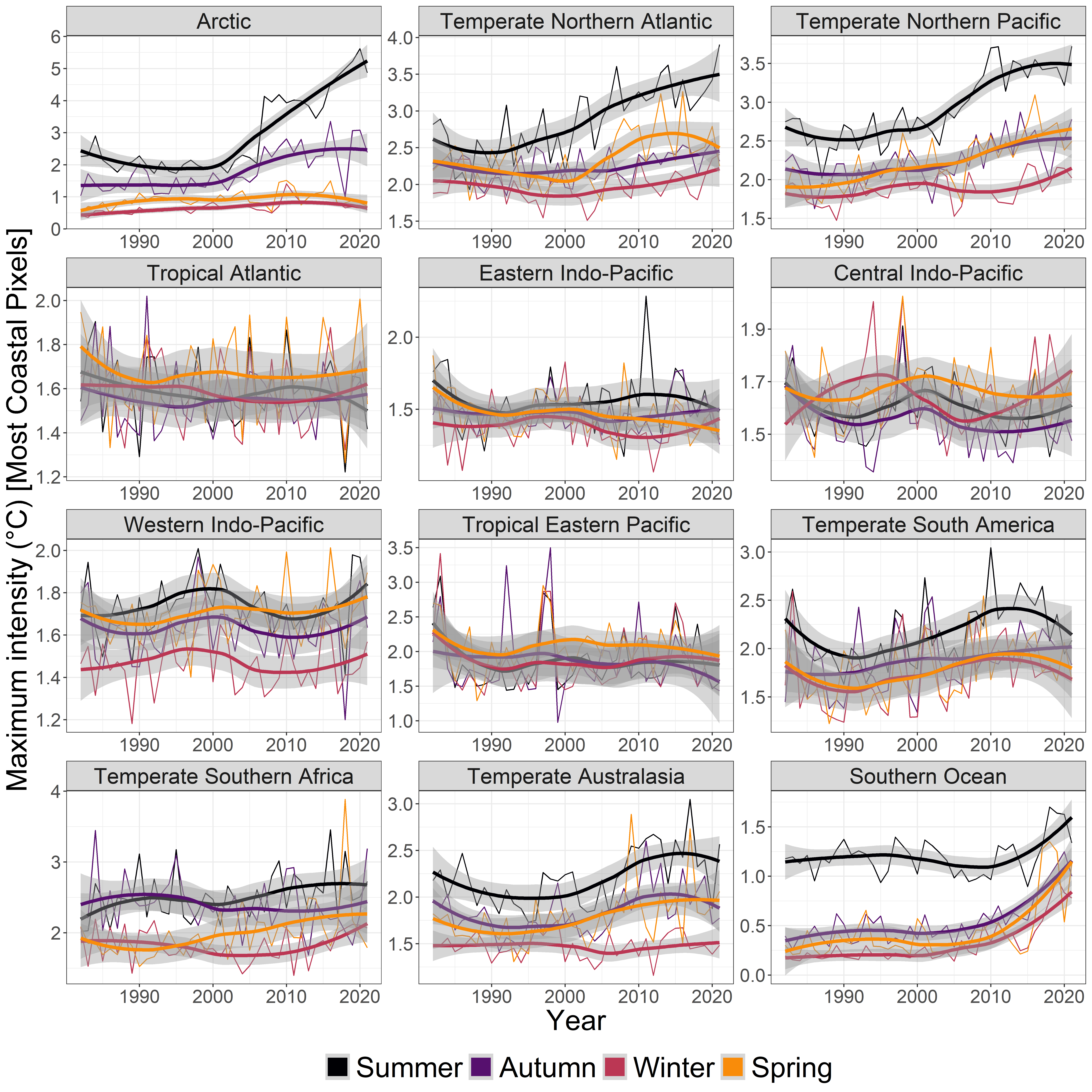


**Figure S13.** Seasonal trends from 1982 to 2021 in the yearly-averaged and pixel-averaged cumulative intensity (°C days) of marine heatwaves per coastal realm and season. Only the most coastal OISST pixels have been used here. See caption of figure 2 for similarities in figure legend.


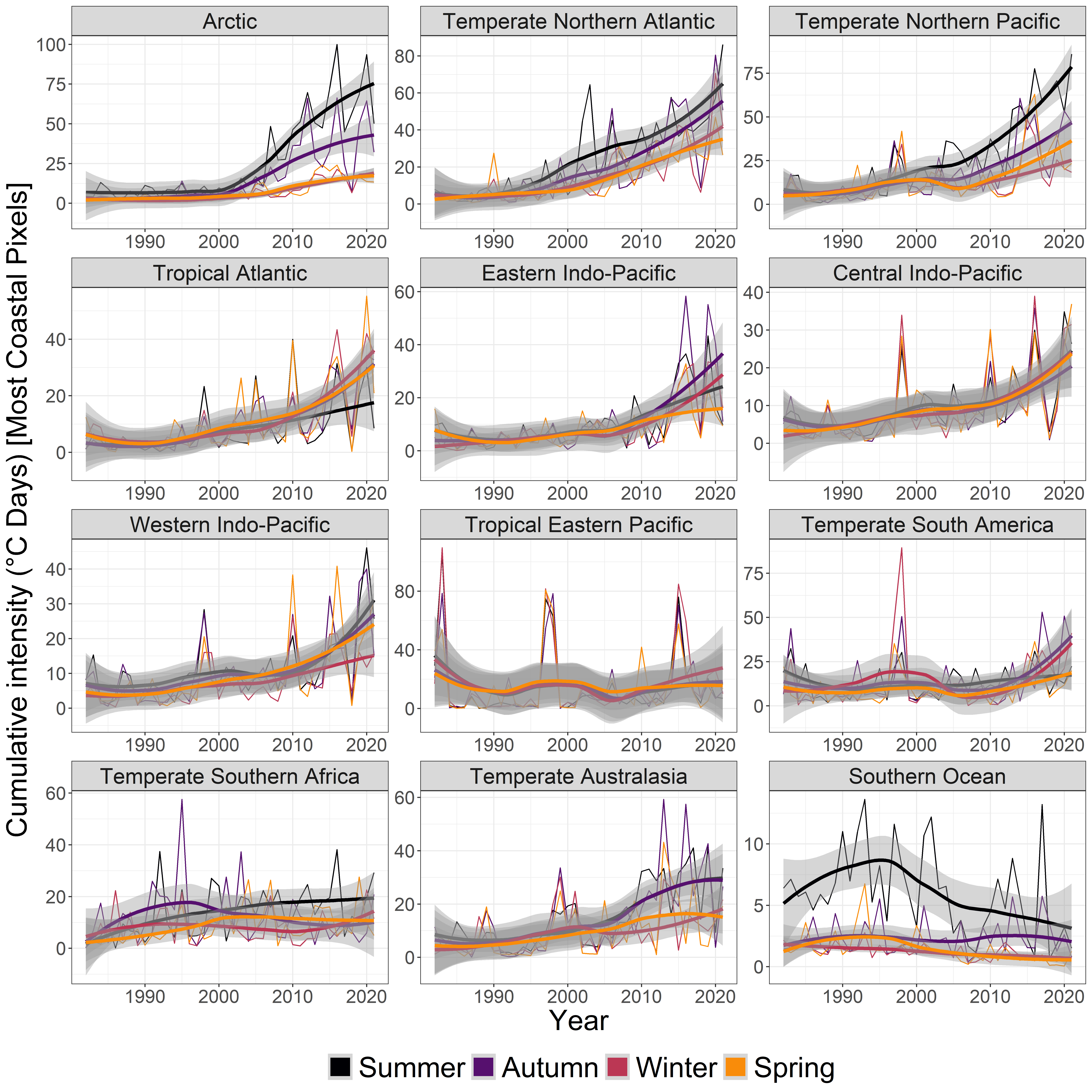


1 Spalding, M. D. *et al.* Marine ecoregions of the world: a bioregionalization of coastal and shelf areas. *BioScience* **57**, 573-583 (2007).
